# Earliest curry in Southeast Asia and the global spice trade 2000 years ago

**DOI:** 10.1126/sciadv.adh5517

**Published:** 2023-07-21

**Authors:** Weiwei Wang, Khanh Trung Kien Nguyen, Chunguang Zhao, Hsiao-chun Hung

**Affiliations:** ^1^ Department of Archaeology and Natural History, Australian National University, Canberra, ACT 2601, Australia.; ^2^ Center for Archaeology, Southern Institute for Social Sciences, Ho Chi Minh City 700000, Vietnam.; ^3^ Department of History (Zhuhai), Sun Yat-sen University, Zhuhai 519082, China.

## Abstract

The global spice trade has played an essential role in world history. However, because of poor preservation conditions, archaeobotanical remains of spices have been limited in archaeological contexts until now. This study reports evidence for spice processing from the archaeological site of Oc Eo in southern Vietnam, an entrepôt of the state of Funan that was occupied during the early centuries CE. Analysis of plant microremains recovered from the surfaces of Oc Eo grinding stone tools thought to be of South Asian origin has identified culinary spices that include turmeric, ginger, fingerroot, sand ginger, galangal, clove, nutmeg, and cinnamon. These spices are indispensable ingredients used in the making of curry in South Asia today. We suggest that South Asian migrants or visitors introduced this culinary tradition into Southeast Asia during the period of early trade contact via the Indian Ocean, commencing about 2000 years ago.

## Teaser

Understanding of the ancient spice trade is expanded through analysis of 2000-year-old processing tools from Vietnam.

## INTRODUCTION

Spices have been highly valued and sought-after since ancient times and have played a key role in building bridges between different cultures (*1*–*3*). South Asia has served as a major source of spices since the Bronze Age, and evidence has shown the movements of turmeric (*Curcuma*), cinnamon (*Cinnamomum*), and black pepper (*Piper nigrum*) from South Asia to the Mediterranean during the second millennium BCE (Before the Common era) (*3*–*5*). By the last centuries BCE and early centuries CE (Common era), historical texts from China, Roman Europe, and India suggest a knowledge of even more exotic spices that originated in Southeast Asia (*6*–*8*).

**Fig. 1.**
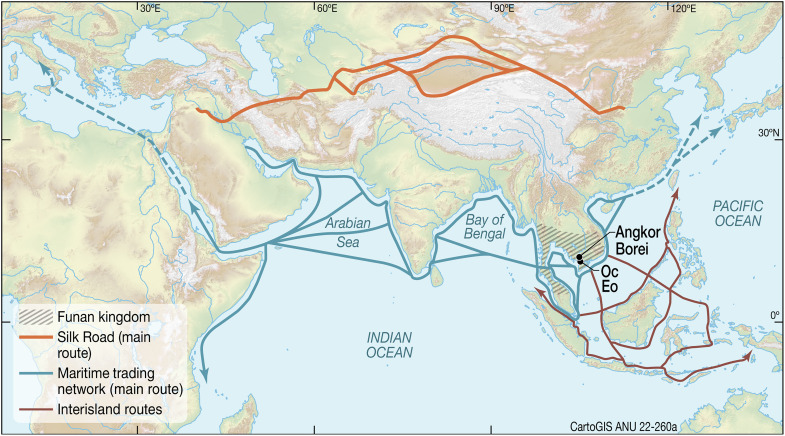
Potential maritime trading networks, the realm of Funan, and the location of Oc Eo. The maritime trading networks and the Silk Road linked the Eurasian continent and the ancient civilizations of the world at least since 2000 years ago. Southeast Asia, such as the trading entrepôt, Oc Eo, is at the crossroads of the ancient maritime trading networks (*78*, *79*).

Southeast Asia played a special role in the spice trade, both as a source of tropical products and as a geographical intermediary between China and the Indian subcontinent (*9*). According to interpretations of Chinese records, the polity of Funan (first to seventh centuries CE) was located at the head of the Mekong Delta (*10*, *11*), from where it could control the Thai-Malay Peninsula and especially the narrow portage presented by the Isthmus of Kra. Two major Funan-era archaeological landscapes have been identified—Angkor Borei in the lower Mekong Valley in southern Cambodia as a potential state capital, and Oc Eo, the focus of this report, downstream at the head of the Mekong Delta in southern Vietnam, as a trading entrepôt (*12*, *13*) (Fig. 1).

In the archaeological inventories from Angkor Borei and Oc Eo, a specific set of stone implements for food preparation has been identified that includes footed grinding slabs and mullers, together with shaped mortars and pestles. These items equate with similar implements found in early historical sites in South Asia, and they appeared in Funan in a context of early cultural contact between the Indian subcontinent and Southeast Asia. These items account for most of the ground stone tools found at Angkor Borei and Oc Eo, and their usage continued during the Dvaravati period in central Thailand, dating between the 7th and 11th centuries CE (*14*–*16*).

The most diagnostic artifact, the footed grinding slab, is traditionally still used for preparing curry paste, and examples have been excavated from South Asian early historic settlements that date between 500 BCE and 300 CE, such as Hastinapur in north India, Pauni and Nagda in central India, and Godawaya and Tissamaharama in Sri Lanka (*16*, *17*). Its abrupt appearance in southern Vietnam, in an indigenous Iron Age context that previously lacked such items, implies the transmission of an exotic cuisine that still exists across both South and Southeast Asia (*18*).

Until now, direct archaeobotanical evidence for a culinary use of spices in both South and Southeast Asia during Iron Age and early historical times has been limited. Some studies in South Asia have reported archaeological presences of black pepper, mustard (*Brassica* spp.), clove (*Syzygium aromaticum*), nutmeg (*Myristica fragrans*), and cardamom (*Elettaria cardamomum*) (*19*–*22*). However, before our investigation, the only evidence from Mainland Southeast Asia came from Angkorian and Post-Angkorian contexts dated between the 11th and 18th centuries CE that had yielded black pepper and long pepper (*Piper longum*) (*23*). In this study, we report on the extraction and identification of starch grains, phytoliths, and pollen grains on the surfaces of a sample of stone processing tools excavated from Oc Eo.

### The site of Oc Eo

**Fig. 2.**
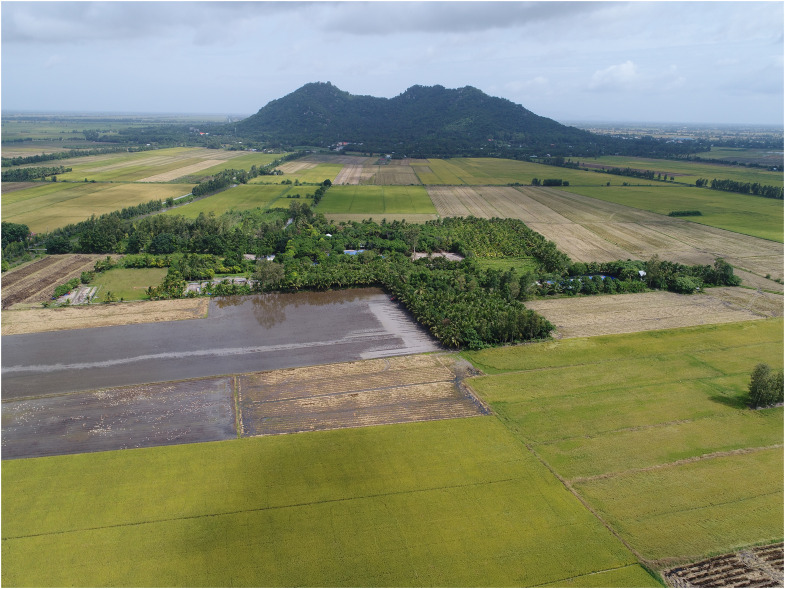
A panoramic view of Mount Ba The from Oc Eo. The Oc Eo complex’s landscape and the vital landmark, Mount Ba The. Currently, the site is covered of rice fields; modern farmers make good use of the ancient canal system to irrigate their rice plantations (Photo source: Khanh Trung Kien Nguyen).

Oc Eo is located on the border between An Giang and Kien Giang provinces on the southwestern side of the Mekong Delta, southern Vietnam. It now lies about 25 km from the sea, within a network of ancient canals (Supplementary Materials, fig. S1). The whole site complex covers an area of some 2,500 ha, including the lower slopes and summit of Mount Ba The and an adjacent floodplain to its southeast that has several natural but low-lying mounds (Fig. 2; Supplementary Materials, Supplementary Text and fig. S2).

**Fig. 3.**
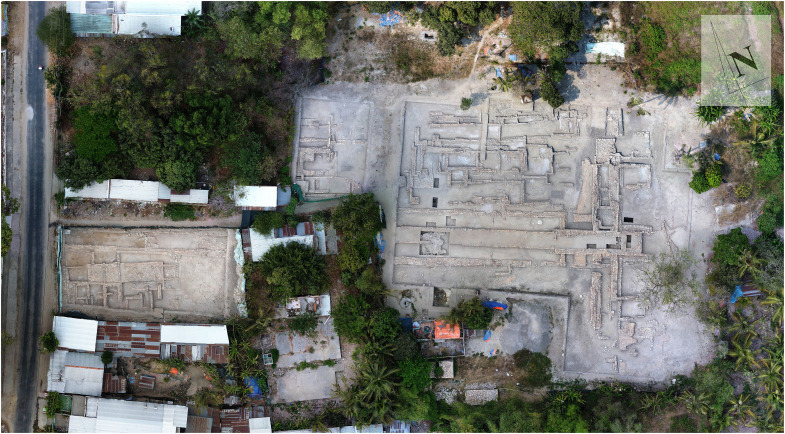
Aerial view of brick foundations at Go Sau Thuan in the Oc Eo complex. These foundations are thought to belong to an entrance work associated with a former Buddhist temple. They date mainly from the sixth to eighth centuries CE (Oc Eo period) but continued in use into the ninth century (Chenla period) (Photo source: Khanh Trung Kien Nguyen).

After the first excavation campaign conducted by French archaeologist Louis Malleret in the 1940s, Oc Eo was recognized as a major overseas trading center of the ancient Funan kingdom. The site also contained Hindu and Buddhist religious monuments and was a center for the processing of metals, glass for jewelry, and pottery (*14*). The 14C (Carbon-14) dates from Oc Eo reveal a major occupation between the first and eighth centuries CE, with the fourth to sixth centuries CE thought to have witnessed the greatest prosperity, when a series of canals, religious monuments, and residential settlements were created (*14*) (Fig. 3; Supplementary Materials, Supplementary Text, figs. S3 to S5, and table S1). A few pieces of Islamic pottery dating from the eighth century represented the final habitation, when the site was abandoned perhaps because of a marine incursion (*14*).

### The data analyzed

**Fig. 4.**
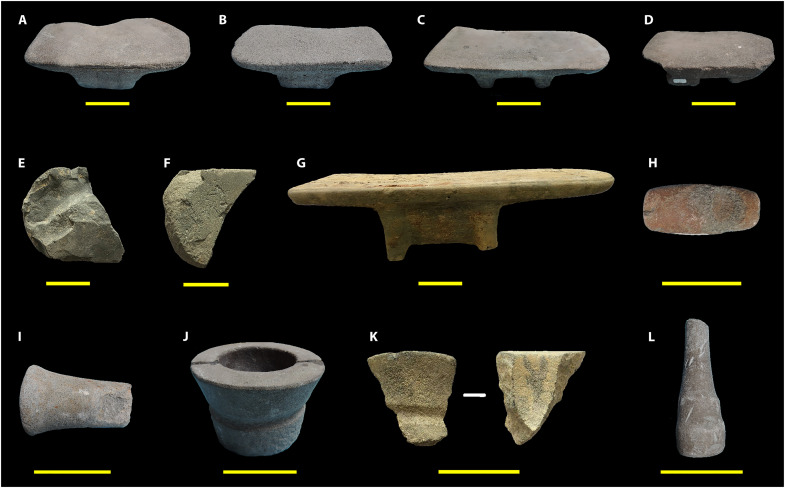
Ancient food preparation equipment reported in this research. (**A** to **D** and **G**) Footed grinding slabs. (**E** and **F**) Fragments thereof. (**H**, **I**, and **L**) Mullers/ pestles. (**J**) Complete mortar. (**K**) Mortar fragment (for details, see Supplementary Materials, table S2). Scale bar, 10 cm.

**Fig. 5.**
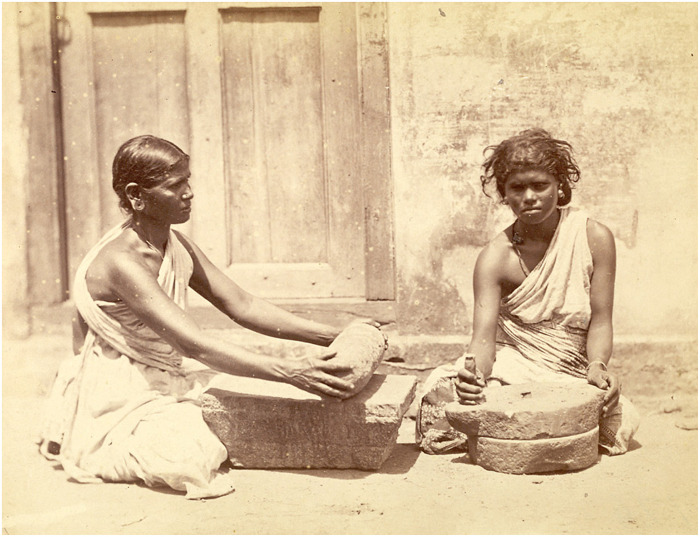
This photograph, shown at the Vienna Universal Exhibition of 1873, shows at left a woman crushing spices with a stone roller on a grinding slab similar to those from Oc Eo (but without separately carved feet). The woman at right is grinding *ragi* (finger millet, *Eleusine coracana,* of late Harappan African origin), a grain cultivated as a food staple in Southern India, between two round millstones. Photo taken at Madras (Chennai) in Tamil Nadu by Nicholas and Curths in c.1870, from the Archaeological Survey of India (Photo source: British Library Board).

This study focuses on microscopic plant remains extracted from 12 food preparation tools found at Oc Eo, illustrated in Fig. 4 and listed in Supplementary Materials, table S2. In South Asia, ground stone processing implements of the types found at Oc Eo are reported from many archaeological sites that date especially to the early historical period (ca. 500 BCE to 300 CE) (*17*). Similar stone tools have remained widely in use in India until recent centuries (Fig. 5).

**Fig. 6.**
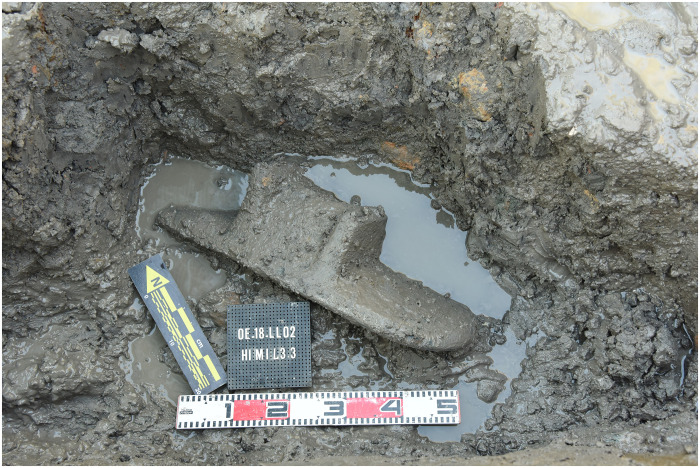
The large footed grinding slab. This footed sandstone grinding slab (sample code: OE18.LLO.B.H1.M1.L3.3), 76 cm long by 31 cm wide, was excavated in 2018. Starch grains of ginger (*Zingiber officinale*), cinnamon (*Cinnamomum* sp.), and nutmeg (*Myristica fragrans*) have been identified on its surface (Photo source: Khanh Trung Kien Nguyen).

At Oc Eo itself, one 14C date of 207 to 326 cal. CE (14C laboratory code: PLD-45555) came from a charcoal sample collected from slightly below our largest grinding slab (sample code OE18.LLO.B.H1.M1.L3.3) (Fig. 6) (Supplementary Materials, fig. S6 and table S1). This date falls well within the occupation span at Oc Eo (*14*, *24*). Presumably, tools of these South Asian types were initially brought to Oc Eo by migrants and later manufactured locally (*16*). More generally, small-scale population movement from South into Southeast Asia is attested as early as the first to third centuries CE by ancient DNA from the Vat Komnou cemetery at Angkor Borei (*25*).

## RESULTS

A total of 717 starch grains were recovered from the surfaces of the 12 studied implements, of which 604 were identifiable to species. The identified specimens were classified into five major types, with Type I (Zingiberaceae) having five subtypes (Ia to Ie). Thus, we recognized a total of eight different spices, together with a presence of rice (*Oryza sativa*) (Table 1) (Supplementary Text; Supplementary Materials, fig. S7 and table S3). Many starch grains show signs of deformation, including broken edges, flat surfaces, loss of lamellae, and weakened extinction crosses, all features that are consistent with damage caused by grinding. Besides the starch grains, phytoliths, and pollens (Supplementary Materials, table S4), other plant tissues such as cells, vessels, and fibers can also aid the identification of ancient spices (Supplementary Text).

**Table 1.** Numbers of starch grains of Types Ia to V (including the five subtypes of Type I) identified from 12 Oc Eo food preparation stone implements. Types I to IV are spices; Type V is rice. UNCLAS, unclassified starch grains.

Number	Artifact code	Tool type	Starch Type										Total
			Ia	Ib	Ic	Id	Ie	II	III	IV	V	UNCLAS	
1	BTAG-2191	Footed grinding slab	3	45		5		1				1	55
2	BTAG-2193	Footed grinding slab	2	1			1	2	7			19	32
3	BTAG-2008-D	Footed grinding slab		1						6			7
4	BTAG-4127-D	Slab fragment	2	2					4			9	17
5	OE.19.GGC.A.H2.L5	Slab fragment	1					2	8	2		13	26
6	OE.19.GGC.A.H1.L8	Footed grinding slab		1				2	10	5		10	28
7	OE.18.LLO.B.H1.M1.L3.3	Footed grinding slab		1					2	3		4	10
8	BTAG-2187-D	Muller	1	1					7	3	22	9	43
9	BTAG-2188-D	Muller		3		3	2		4		29	14	55
10	BTAG-2019-D	Mortar	115	28	31	29	37	119	14	5	12	17	407
11	OE.19.LLO.B.H3.b4.L2.7	Mortar fragment							5	13		15	33
12	BTAG-3147-D	Pestle	1					1				2	4
**Total**			125	83	31	37	40	127	61	37	63	113	717
**Total %**			17.43%	11.58%	4.32%	5.16%	5.58%	17.71%	8.51%	5.16%	8.79%	15.76%	100%

**Fig. 7.**
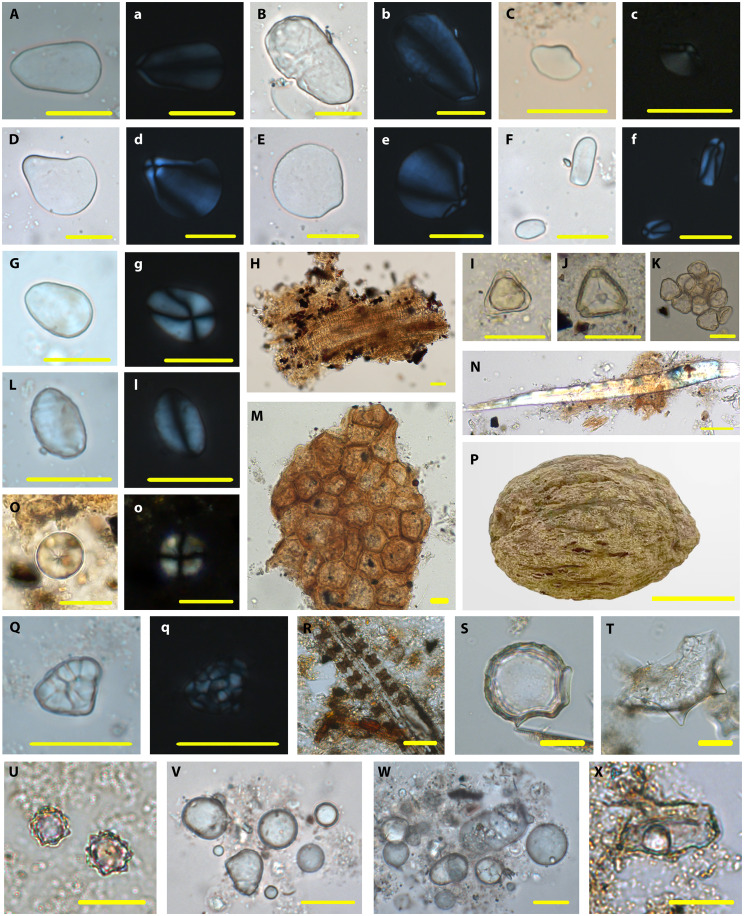
Ancient plant remains recovered from Oc Eo. (**A** to **g**, **L** and **l**, **O** and **o**, and **Q** and **q**) Starch grains under polarized and bright-field light. (**H** to **K**, **M** and **N**, **P**, and **R** to **X**) Other plant remains. (A) and (a), (B) and (b) Type Ia, *Curcuma longa*. (C) and (c) Type Ib, *Z. officinale*. (D) and (d) Type Ic, *Boesenbergia rotunda*. (E) and (e) Type Id, *Kaempferia galanga*. (F) and (f) Type Ie, *Alpinia galanga*. (G) and (g) Type II, *Syzygium aromaticum*. (H) Fragments of spiral vessel. (I) to (K) Myrtaceae pollen. (L) and (l) Type III, *Cinnamomum* sp. (M) Cork cells. (N) Fiber. (O) and (o) Type IV, *M. fragrans*. (P) Nutmeg. (Q) and (q) Type V, *Oryza sativa*. (R) Rice parallel bilobate phytolith. (S) Rice bulliform phytolith. (T) Rice double-peaked phytolith. (U) Spheroid echinate. (V) and (W) Spheroid psilate. (X) Volcaniform phytolith. Scale bar in (P) is 1 cm, and all others are 20 μm.

**Fig. 8.**
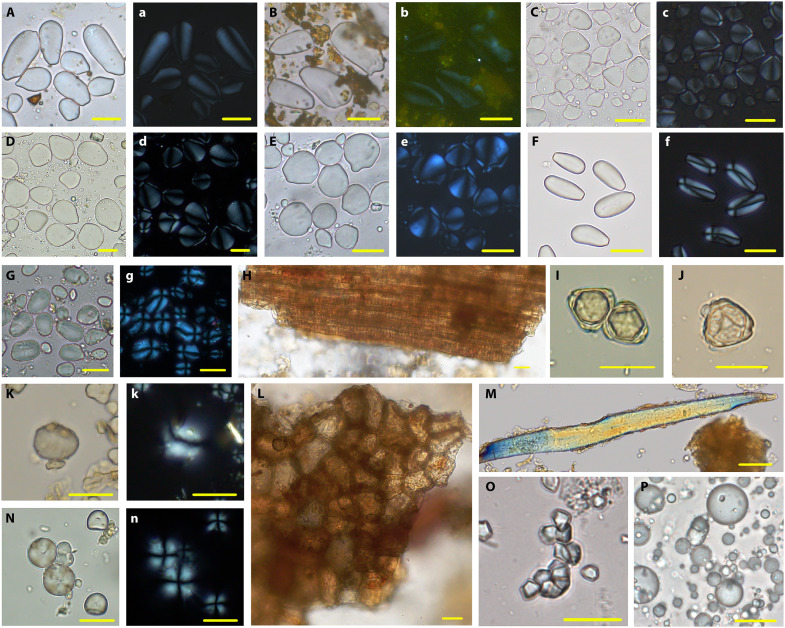
Modern references relevant to this study. (**A** to **g**, **K** and **k**, and **N** and **n**) Starch grains under polarized and brightfield light. (**H** and **J**, **L** and **M**, and **O** and **P**) Other plant remains. (A) and (a) *C. longa*. (B) and (b) Turmeric powder. (C) and (c) *Z. officinale*. (D) and (d) *B. rotunda*. (E) and (e) *K. galanga*. (F) and (f) *A. galanga*. (G) and (g) *S. aromaticum*. (H) Spiral vessels from clove powder. (I) and (J) Clove pollen. (K) and (k) *Cinnamomum verum*. (L) Cork cell from Ceylon cinnamon. (M) Fiber from Ceylon cinnamon. (N) and (n) *M. fragrans*. (O) *O. sativa* subsp. *japonica*. (P) Coconut spheroid psilate. Scale bar, 20 μm.

Among the five main types [Types I (Ia to Ie), II, III, V, and VI] of identified starch (*n* = 604), 514 granules could be identified as coming from eight different identified spices. Of these, 316 belonged to Zingiberaceae species (Type I variants; Table 1), identifiable especially by their extremely eccentric protruding hila. Starch grains from turmeric (*Curcuma longa*; Type Ia) account for the largest proportion of these and are elongated and ovate in shape with an eccentric protruding hilum (Figs. 7, A and a, B and b, and 8, A and a). Many show broken features caused by grinding, like starch granules found in modern curry powder (Fig. 8, B and b). Eighty-three small oval starch grains of Type Ib with protruding hila can be identified as coming from ginger (*Zingiber officinale*) (Figs. 7, C and c, and 8, C and c). Thirty-one starch grains of Type Ic share a similar shape with ginger but have larger sizes that are more comparable with fingerroot (*Boesenbergia rotunda*) (Figs. 7, D and d, and 8, D and d). The starch grains from sand ginger (*Kaempferia galanga*; Type Id) are quite unique, with a sub-rounded shape and a highly eccentric and convex hilum (Figs. 7, E and e, and 8, E and e). Forty narrow oval starch grains of Type Ie very closely resemble those from galangal (*Alpinia galanga*) (Figs. 7, F and f, and 8, F and f).

In addition to Zingiberaceae, starch grains from three other plant families were found on the Oc Eo processing tools. They include clove seeds (Type II) that have ovate starch grains with an eccentric hilum and visible lamellae (Figs. 7, G and g, and 8, G and g). Several groups of spiral vessels and more than 300 pollen grains are also thought to be from cloves in terms of their metrical features, and these have been identified on a newly excavated grinding slab (OE.19.GGC.A.H2.L5/H1.L8; and see Figs. 7, H to K, and 8, H to J; and details in the Supplementary Text).

Sixty-one starch grains of type III (Figs. 7, L and l, and 8, K and k), as well as cork cells (Figs. 7M and 8L) and fibers (Figs. 7N and 8M), share high morphological similarity with cinnamon (*Cinnamomum* sp.). Thirty-seven starch grains of Type IV can be identified as nutmeg (*M. fragrans*); these are characterized by round shapes and radiating or linear fissures (Figs. 7, O and o, and 8, N and n). Notably, a large number of macroremains of nutmeg were found in Oc Eo, and one seed was directly dated to 120 to 248 cal. CE (Beta-629719; 94.3% probability). Unexpectedly, nearly 2000 years later, it still yielded a nutmeg aroma (Fig. 7P).

Starch grains (Type V) (Figs. 7, Q and q, and Fig. 8O) and phytoliths (Fig. 7, R to T) of rice (*O. sativa*) were also found in our samples, together with the spheroid echinate phytoliths from palms (Fig. 7U). Notably, substantial quantities of spheroid psilate were found on the mortar BTAG-2019 (Fig. 7, V and W). They were probably derived from coconut (*Cocos nucifera*), according to comparisons with modern reference samples (Fig. 8P). Eleven volcaniform phytoliths of *Musa* (Fig. 7X) were found on BTAG-2191.

## DISCUSSION

Using stone pounding and grinding tools to process edible plants is a worldwide practice that can be traced deeply into prehistory. The methodology of analyzing microremains that we have used in this report has been applied widely elsewhere, for instance in identifying the processing of wheat and barley in the Middle East (*26*), foxtail and broomcorn millet in northern China (*27*), maize and manioc in central America (*28*, *29*), and taro and yam in the subtropical and tropical Asia-Pacific (*30*, *31*). Through our analyses of the microremains found on the surfaces of the grinding tools from Oc Eo, a number of economic and staple plants, including spices, have been identified in contexts dating between 2000 and 1300 years ago. The artifacts analyzed correspond with archaeological and traditional Indian spice grinding tools, designed to release the flavors and tastes that characterize different spices.

### Turmeric (*C. longa*)

**Fig. 9.**
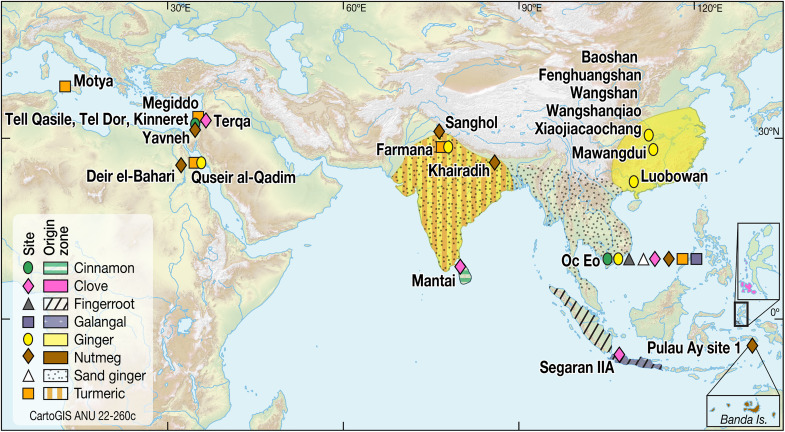
The archaeological sites discussed in this study and possible origin regions for the spices extracted as starch grains from the Oc Eo implements. Eight types of spices were extracted from the ancient stone tools of Oc Eo. The possible origins of these specific spices include the Moluccas in East Indonesia, India, Sri Lanka, and other candidates such as Java in central Indonesia (see Supplementary Materials, table S5).

Turmeric most probably originated in South Asia, where the greatest diversity of *Curcuma* species and the earliest turmeric remains occur (*32*) (Fig. 9). Starch grains from turmeric have been extracted from Harappan (2600 to 2200 BCE) cooking pots and human teeth found at Farmana in Haryana (*33*). Turmeric was involved in trading systems between South Asia and the Mediterranean by the middle of the second millennium BCE, indicated by the turmeric protein identified in the dental calculus of an individual from Bronze Age Megiddo in Israel (*3*). Furthermore, turmeric is mentioned in Assyrian medical texts from Ashurbanipal’s library at Nineveh (seventh century BCE; *3*, *34*). In Southeast Asia, turmeric was perhaps introduced to produce yellow dye for the robes of Buddhist monks (*35*, *36*). Archaeobotanical findings of turmeric were unknown in Southeast Asia before this study, and the large quantity of turmeric starch found at Oc Eo underlines the culinary influence there of Indian culture.

### Ginger (*Z. officinale*)

Ginger originated in the Indo-Malaysian region, but the primary domestication center remains obscure (*37*). China and India have been suggested as two major cultivation centers and suppliers of ginger for trade since ancient times (*38*) (Fig. 9). China has the oldest written record for its presence, from the Analects of Confucius written in the Warring States period (475 to 221 BCE) (*39*). However, ginger starch has been recovered from the Harappan site of Farmana in India (*33*). In China, more than 78 pieces of ginger have been found from seven ancient burials from the late Warring States period to early Western Han Dynasty (300 to 160 BCE) (*40*) (Supplementary Materials, table S5). Ginger was well known to the West in the first century CE, and references to its medicinal and spice usages occur in Persian, Arabic, Greek, and Roman texts (*6*, *34*).

### Other ginger plants

The other edible Zingiberaceae species found at Oc Eo—galangal (*A. galanga*), fingerroot (*B. rotunda*), and sand ginger (*K. galanga*)—are all native to tropical Asia and widely cultivated today in Southeast Asia. Their precise origins are uncertain (*41*, *42*), but it is possible that galangal originated in Java (*38*), fingerroot in Sumatra and Java (*2*, *43*), and sand ginger less precisely in either South or Southeast Asia (*44*) (Fig. 9). So far, traces of these ginger species have been absent in archaeological records, except for a seed of *Alpinia* sp. identified from the temple of Ta Prohm at Angkor, with a radiocarbon date that falls within ca. 8th through 10th centuries CE (*23*).

Thus far, no other finds of galangal have been reported from the archaeological record, but seeds of the related shell ginger (*Alpinia* cf. *zerumbet*) have been recovered from Kantharodai (ca. 200 BCE) and Kirinda (ca. 260 to 900 CE) in Sri Lanka (*45*). Lesser galangal (*Alpinia officinarum*) is reported from the Mawangdui Western Han Tomb (second century BCE) in Hunan province, China (*46*). The Roman author Apicius used galangal in his recipes, and galangal figured in early trade from Asia to Europe (*47*). Medicinal uses of galangal are recorded in Sanskrit texts that date back to 600 CE (*6*). The other two Zingiberaceae species—fingerroot (*B. rotunda*) and sand ginger (*K. galanga*)—are used today as seasonings and herbal medicines and also have a long history of utilization in Southeast Asia (*48*, *49*).

### Clove (*S. aromaticum*)

Clove trees are native to the volcanic islands of Maluku Utara (northern Moluccas), often termed the “Spice Islands” (*50*) (Fig. 9). The common term “clove” refers to the clove bud, but the clove seed, termed “chicken-tongue spice” in Chinese texts, was also a traditional spice and medicine. Several historical documents suggest that cloves reached China, India, and Rome during the late centuries BCE to early CE. For instance, according to the *Han Guan Yi* (Etiquettes of the Officialdom of the Han Dynasty) completed ca. 200 CE, the Han Dynasty court had a rule that ministers should suck cloves to sweeten their breath before speaking to the emperor (*51*). Cloves are mentioned in the Ramayana, an Indian epic written between 350 BCE and 1 CE, and are reputed according to Pliny’s Natural History (70 CE) to have been known in Rome (*22*, *52*).

Before this study, the earliest potential evidence for cloves, dated to ca. 1700 BCE, came from the Middle Bronze Age site of Terqa in Syria, but the identification remains controversial (*53*, *54*). A carbonized clove was excavated from the deposit of ca. 900 to 1100 CE in the ancient port of Mantai in Sri Lanka, South Asia (*21*). In West Java, a clove was recovered from the lower waterlogged layer (second century BCE to third century CE) at the Segaran IIA site at Batujaya (*55*). Notably, a few ceramic shards from Batujaya resemble pottery at Oc Eo and suggest contact (*55*). It is unclear whether cloves were directly obtained from source plantations in the Moluccas or whether the tree was planted in islands to the west of the Moluccas (such as Java) from an early date. Clove plantations occur in many Indonesian islands today.

### Nutmeg (*M. fragrans*)

The earliest direct evidence of nutmeg comes from the Banda islands, where it originated (*50*, *56*) (Fig. 9). Potsherds excavated from Neolithic layers (ca. 1500 to 300 BCE) in Pulau Ay site 1 have produced starch grains from nutmeg (*56*). Far to the west, claims for nutmegs at Deir el-Bahari (16th to 14th centuries BCE) in Egypt and at Yavneh (ca. 9th century BCE) in Israel remain unsubstantiated by definite identification (*57*, *58*). More convincingly, carbonized nutmeg from Khairadih in eastern Uttar Pradesh (Ganges Valley) has been dated to an early phase of the Northern Black Polished Ware Culture (700 to 200 BCE) (*22*), and to the Kushan period from a fire altar at Sanghol (100 to 300 CE) in Punjab (*22*) (Fig. 9). A document termed the Charaka Samhita, an Ayurvedic compendium dating from the first century CE with a particular focus on general and internal medicine, mentions nutmeg, mace (the seed covering of nutmeg) and cloves (*22*).

Nutmeg seeds were recovered during the recent excavations at Oc Eo. One is directly dated to 120 to 248 cal. CE (94.3% probability), and this is so far the oldest nutmeg specimen in Mainland Southeast Asia.

### Cinnamon (*Cinnamomum* sp.)

Cinnamon is one of the most ancient spices in history. It can be obtained from several tree species in the genus *Cinnamomum* (*59*). “True cinnamon” usually refers to Ceylon cinnamon (*Cinnamomum verum*) and is native to Sri Lanka (*60*) (Fig. 9). Other cinnamons, more precisely called cassia, come from four species; Chinese cinnamon (*Cinnamomum cassia*), Saigon cinnamon (*Cinnamomum loureiroi*), Indonesian cassia (*Cinnamomum burmannii*), and Indian cassia (*Cinnamomum tamala*) (*61*, *62*).

Cinnamon has been involved in global trade at least since 2000 years ago. Egyptian records suggest its use for embalming mummies (*63*) in the second millennium BCE, but the details are questionable because of lack of direct identification (*58*). Cinnamon has been reported from three sites in Israel dated between the 11th and late 10th centuries BCE (*57*), and a damaged flower dated to the 7th century BCE found at Heraion on the Greek island of Samos was probably *C. cassia* (*64*). Cinnamon is also mentioned in the Old Testament and Pliny’s Natural History (*65*, *66*, *67*).

The microremains from Oc Eo show similar morphologies to Ceylon cinnamon from Sri Lanka. The discovery of cinnamon residues on the Oc Eo tools indicates its long-term utilization in Southeast Asia.

### Other nonspice plants

Apart from spices, carbonized rice grains are common at Oc Eo, as also at Angkor Borei, where rice husks occur in earthenware pottery and bricks (*68*). The starch grains and phytoliths of rice from Oc Eo support the existence of a well-developed agricultural system as the basis for the Funan economy, echoing historical records (*69*). The canal systems recorded by Malleret in ancient Oc Eo (Supplementary Materials, fig. S1) would have provided irrigation water, just as such canals do today (*69*).

Palm products were also economically important in ancient Southeast Asia, for building houses, providing fibers for rope, and boat construction and in cooking and for alcoholic beverages (*21*). The legend of Funan in the Chinese Book of Liang (sixth century CE) records procedures for making palm wine in Southeast Asia, including the cutting of flowers, collection of sap, and fermentation in pots to make the wine.

This study of microremains on stone grinding and pounding tools from Oc Eo has revealed that Funan people used spices from several species indigenous to South and Southeast Asia, including turmeric, ginger, galangal, sand ginger, fingerroot, clove, nutmeg, and cinnamon. Species in the family Zingiberaceae were especially prominent. All of these spices can be used as ingredients for making curry (Fig. 5), and some, such as cinnamon, nutmeg, and clove, might have been imported to Oc Eo from distant locations in South Asia and eastern Indonesia (Fig. 9). However, the possibility that these species were reproduced from seed stock in places far from their homelands, at dates close to their entry into culinary repertoires, needs always to be borne in mind.

Perhaps the history of curry began more than 4000 years ago in Harappan Pakistan and India, where starch grains of turmeric, ginger, eggplant, and mango have been found attached to human teeth and in cooking pots (*33*). Today, curry is still popular in Southeast Asia (*70*), and the ingredients recovered from Oc Eo are matched more closely in modern Southeast Asia than in South Asia in being mixed with endemic spices and thickened by coconut milk. For example, galangal is a common component of curry pastes in Southeast Asia but is seldom used in Indian curry (*18*), likewise fingerroot and sand ginger in Thai curry (*71*).

Considering multiple lines of evidence, particularly the newly found spices from this study and their association with Indian-style preparation tools, we may conclude that curry recipes arrived in Southeast Asia with South Asian traders and migrants as contacts intensified during the early centuries CE. This study thus bolsters our knowledge about how South Asian cultures influenced the formation of early Southeast Asian cuisines, especially in light of the dynamic role of ancient Oc Eo, in southern Vietnam, within global maritime trade networks.

The global spice trade has linked cultures and economic systems in Asia, Africa, and Europe since Classical times. Our findings provide direct evidence for a role in this trade of the ancient port city of Oc Eo, by at least 2000 to 1800 years ago. Oc Eo likewise maintained contacts westward with the Indian subcontinent and, more distantly, the Mediterranean world. Our findings also highlight the importance of the Moluccas as the ultimate source region for cloves and nutmegs.

## MATERIALS AND METHODS

In 2018 and 2019, we selected 40 stone implements for study from the collections made by several excavations at the sites of Go Giong Cat, Lung Lon, and Go Sau Thuan within the Oc Eo archaeological complex. They were stored in the An Giang Provincial Museum and the Oc Eo Archaeological Field Station. The implements selected were initially cleaned with distilled water and then shaken separately in an ultrasonic water bath for 5 min. Heavy liquid solutions of LST (lithium heteropolytungstate) at specific densities of 1.9 and 2.35 were added to isolate the starch grains, phytoliths, and other microfossils. Among the 40 stone implements, 12 produced good samples of starch remains, while the rest produced less than five starch grains each.

The starch grain samples were then each mounted on a glass slide in a solution of 10% glycerine and 90% water and scanned under an Olympus microscope at magnifications of ×200 and ×400 with both white and cross-polarized light. The phytolith samples were mounted on slides with Canada Balsam and scanned at ×400 magnification. The starch grains, phytoliths, and other microbotanical remains such as pollen grains, vessels, and fibers were counted, photographed, and measured with regard to their morphological characteristics.

The ancient starch identifications are based on comparisons with the starch grains of more than 200 Asian species (*72*). In addition, comparative samples were prepared from 26 purchased modern spice varieties and other economic plants (Supplementary Materials, table S6). Pharmacognostic studies were consulted for those spices used in traditional herbal medicines that had their microscopic characters recorded (*73*, *74*).

Phytoliths were identified following previously published references and criteria (*75*, *76*) and named and classified according to the International Code for Phytolith Nomenclature (ICPN 2.0) (*77*). After analyzing and comparing morphological features within each botanical category analyzed, certain specific and clearly defined characteristics were selected as classification indicators for discrimination between different spice species. The results were then checked against purchased samples of modern mixed spice powder.

## Supplementary Materials

### This PDF file includes:

Supplementary Text Figs. S1 to S7 Tables S1 to S6 References

## Notes

View/request a protocol for this paper from *Bio-protocol*.
