# Supplementary material for: Earliest curry in Southeast Asia and the global spice trade 2000 years ago
Source: Sci Adv. 2023 Jul 21;9(29):eadh5517. doi: 10.1126/sciadv.adh5517 (PMC10361603; doi:10.1126/sciadv.adh5517)
Supplement: Supplementary file 1 — Supplementary Text Figs. S1 to S7 Tables S1 to S6 References [file sciadv.adh5517_sm.pdf]

Science Advances
AAAS

Supplementary Materials for

Earliest curry in Southeast Asia and the global spice trade 2000 years ago

Weiwei Wang *et al.*

Corresponding author: Khanh Trung Kien Nguyen, nktk1979@gmail.com; Hsiao-chun Hung, hsiao-chun.Hung@anu.edu.au

Sci. Adv. **9**, eadh5517 (2023)
DOI: 10.1126/sciadv.adh5517

This PDF file includes:

Supplementary Text
Figs. S1 to S7
Tables S1 to S6
References

Supplementary Text

Further details of Oc Eo and its role in the maritime trading network of early historical Southeast Asia

Oc Eo's existence was noted first by French colonial engineer Pierre Paris when he took aerial photographs of the region in 1931 and 1941 (*69*). In 1944, French archaeologist Louis Malleret conducted field survey and the foundational archaeological campaign in Oc Eo. Malleret reported 300 archaeological sites, many with brick monuments, statuary, architectural pieces, pottery, beads, intaglios, coins, and many other precious goods (*69, 80, 81*). From 1975 to the present, the investigations, surveys and excavations by local Vietnamese archaeologists and collaborative researchers in Oc Eo and adjacent areas have continuously refreshed our knowledge the social, cultural and political contexts of the Oc Eo culture and its dynamic role in maritime trade (*82*).

Oc Eo is located within a network of ancient canals that criss-cross the flat terrain of the Mekong Delta. The main canal, about 110km in length, connected Oc Eo with Angkor Borei to the north and stretched onwards across the Mekong Delta into the Ca Mau Peninsula (*83*) (fig. S1). Other waterways and canals flowed through the "ancient Oc Eo town" defined by Malleret. These waterways thus connected Oc Eo with the outside world and made it into an important transit port of Funan.

The archaeological region of Oc Eo covers an area of some 2,500 ha, and includes two adjacent areas, these being the lower slopes and summits of Mount Ba The, and the floodplain to its south that contains several low-lying mounds, including the eponymous site named Go (mound) Oc Eo (*69*) (fig. S2). On the eastern flank of Mount Ba The, the Linh Son group of religious foundations occupies an area of about 800 by 400 m (fig. S2). In Go Oc Eo, residential areas were distributed along canals, and nearly 70 wooden stakes from former stilt dwellings have been excavated in waterlogged deposits at Lung Lon B (fig. S3). The artifacts found here were mainly occupation and economic refuse, including potsherds and artifacts of stone, terracotta, wood, metal and glass, with varied food remains (e.g., fish bones, animal bones, tree nuts).

The rich collections of imported artifacts discovered by Malleret show that Oc Eo had strong and diverse external trade relations with the Roman, Byzantine, Islamic, South Asian, and Chinese spheres of influence that spanned the 2nd to 8th centuries CE (*84*). Striking examples include coins and medallions of Roman inspiration, a gold ring decorated with a symbol of Nandi (the bovine mount of Shiva), an Eastern Han bronze mirror and Wu Zhu coins, and gold beads of possible Mediterranean origin (2nd-3rd centuries CE) (*14*) (fig. S4).

Details of the starch grain identifications

1. Spices

(1) Turmeric (*Curcuma longa*)

Starch grains from turmeric (Type Ia) account for the largest proportion of the Zingiberaceae. They are elongated and ovate in shape with an eccentric protruding hilum (Fig. 7, A–a, B–b; Fig. 8, A–a). Turmeric starch grains are the largest in size among our

starch findings, ranging in length from 12.69μm to 45.54μm, with a mean of 25.4±6.37μm. Given that turmeric usually is consumed as powder, we investigated the morphology of modern turmeric powder for comparison. We noted that the ancient grains, like the modern turmeric powder, commonly showed flat surfaces, loss of lamellae, and highly weakened extinction crosses. These were due to damage caused by grinding activities (Fig. 8, B–b).

(2) Ginger (*Zingiber officinale*)
Eighty-three small oval starch grains (Type Ib) with protruding hila are identified (Fig. 7, C–c; Fig. 8, C–c) as from ginger. Their most noticeable feature is that the widths of about one-third of the sample are larger than their lengths, with 90% being under 20μm in length (fig. S7). This length-to-width ratio separates ginger from most other Zingiberaceae.

(3) Fingerroot (*Boesenbergia rotunda*)
Thirty-one starch grains from fingerroot (Type Ic) exhibit a triangular-ovate shape with a protruding hilum (Fig. 7, D–d; Fig. 8, D–d). Their lengths range from 19.1μm to 40.98μm. Although the shapes of some fingerroot starch grains overlap with those of ginger, 80% of the long axis diameters are larger than 20μm. Thus, the starch grains from these two species mostly can be distinguished (fig. S7).

(4) Sand ginger (*Kaempferia galanga*)
The shape of this starch grain (Type Id) is sub-rounded, with a highly eccentric and convex hilum (Fig. 7, E–e; Fig. 8, E–e). Lengths range from 12.18μm to 31.69μm, with a mean of 20.23±4.78μm.

(5) Galangal (*Alpinia galanga*)
Galangal starch grains (Type Ie) are narrow and oval with an eccentric center, and the cross arm is curved under polarizing light (Fig. 7, F–f; Fig. 8, F–f). This starch type is distinct in the ginger family.

(6) Clove (*Syzygium aromaticum*)
Starch grains from cloves (Type II) are ovate with an eccentric hilum and visible lamellae (Fig. 7, G–g; Fig. 8, G–g). More than 300 pollen grains that share typical features with the Myrtle family have also been extracted from two newly excavated grinding slabs from Oc Eo (Fig. 7, I–K; Fig. 8, I–J). These are consistent with *Syzygium* in size, metrics and surface features (*85, 86, 87*). These pollen grains originated from clove buds.

(7) Cinnamon (*Cinnamomum* sp.)
Sixty-one ovate starch grains (Type III) with an open or slit-shaped hilum and visible lamellae resemble samples extracted from cinnamon powder (*Cinnamomum* sp.) (Fig. 7, L–l; Fig. 8, K–k). Cinnamon cork cells and fibers are also distinctive and three fragments of cinnamon cork cell exist in our samples (Fig. 7, M; Fig. 8, L). The fibers in modern cinnamon show two ends in blue and a middle zone in yellow under cross-polarized light; the same kind of fiber is found in our samples (Fig. 7, N; Fig. 8, M).

(8) Nutmeg (*Myristica fragrans*)

Thirty-seven Type IV starch grains are characterized by their round shape, with a mean size of 11.31± 3.09μm, open hilum, radiating or linear fissures, and extinction crosses with slightly bent arms (Fig. 7, O–o). They resemble nutmeg starch (*Myristica fragrans*) (Fig. 8, N–n), and this shape is not associated with other spices.

2. Other plants

(1) Rice (*Oryza sativa*)

More than sixty small Type V starch grains with polygonal shapes, centric hila, and straight cross arms share typical features with rice (Fig. 7, Q–q; Fig. 8, O). In addition, phytoliths from rice leaves, stems, and husks were recovered in this study (Fig. 7, R–T), indicating local production and consumption of rice. These observations correspond with historical accounts that Funan people relied on rice agriculture for their fundamental subsistence.

(2) Palms (Arecaceae)

Although spheroid echinate phytoliths from palms were found in the ancient samples analyzed (Fig. 7, U), actual starch grains from palms are absent. This suggests that palms were not targeted food plants in Oc Eo. Of special note, however, substantial quantities of spheroid psilate were discovered on a mortar (BTAG-2019) that also carried starch grains of gingers and other spices. These are spherical or ovate, of varied size, and present erosion holes on their surfaces (Fig. 7, V–W). Spheroid psilate arises as a vesicular infilling of epidermal and parenchyma cells in the foliage and reproductive organs of a wide range of Arecaceae, Poaceae, Cyperaceae, and gymnosperm species (*77*). In our modern reference collection, it could be observed in the seeds of species with high oil contents, such as mustard, cumin and coconut. The spheroid particles from coconut (*Cocos nucifera*) are especially comparable with our findings (Fig. 8, P).

(3) Banana (*Musa* sp.)

Eleven volcaniform phytoliths derived from *Musa* sp. (Fig. 7, X) were found on specimen BTAG-2191, indicating exploitation of bananas.

Provenances and Permissions

The 40 analyzed stone implements were collected by the An Giang Provincial Museum before 2018 (n=20), and excavated between 2017 and 2019 by field teams from The Center for Archaeology, Southern Institute for Social Sciences, Ho Chi Minh City, led by Khanh Trung Kien Nguyen (n=20) (*14*). The sites excavated were Go Giong Cat, Lung Lon, and Go Sau Thuan, all located within the Oc Eo archaeological complex. Radiocarbon dates related these artifacts have been published by Khanh Trung Kien Nguyen and colleagues (*24*). In 2018 and 2019, the four authors selected the 40 specimens used in this study from the collections stored in the An Giang Provincial Museum and the Oc Eo Archaeological Field Station, southern Vietnam. Access to these materials was granted by the Center for Archaeology, Southern Institute for Social Sciences.

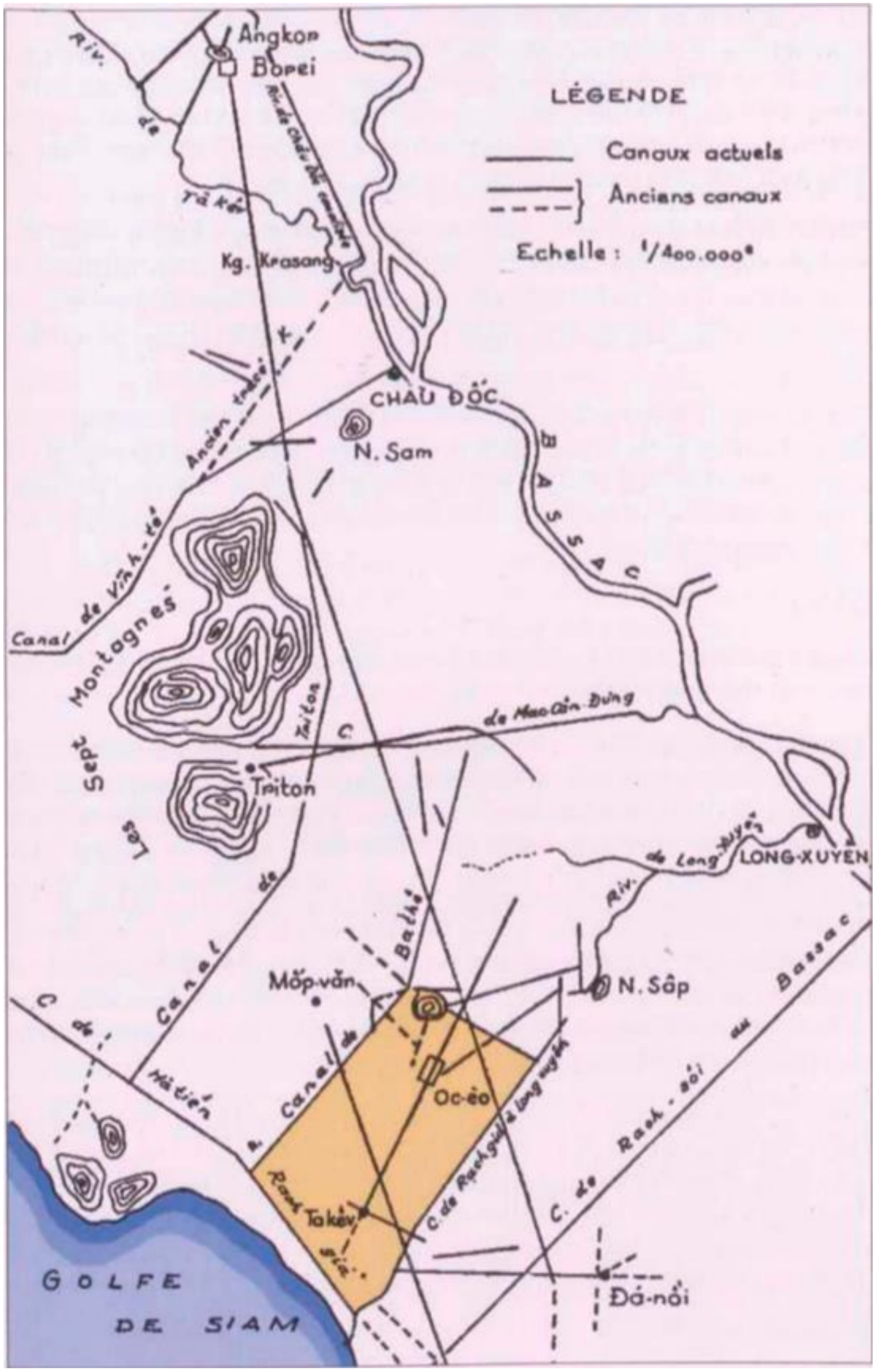

Fig. S1. Louis Malleret's plan of Angkor Borei and Oc Eo.
The plan shows the locations of Mount Ba The (*Les Sept Montagnes*), the Bassac distributary of the Mekong River, the reconstructed canal system of the Mekong Delta, and the locations of the major archaeological sites of Angkor Borei and Oc Eo (*80*).

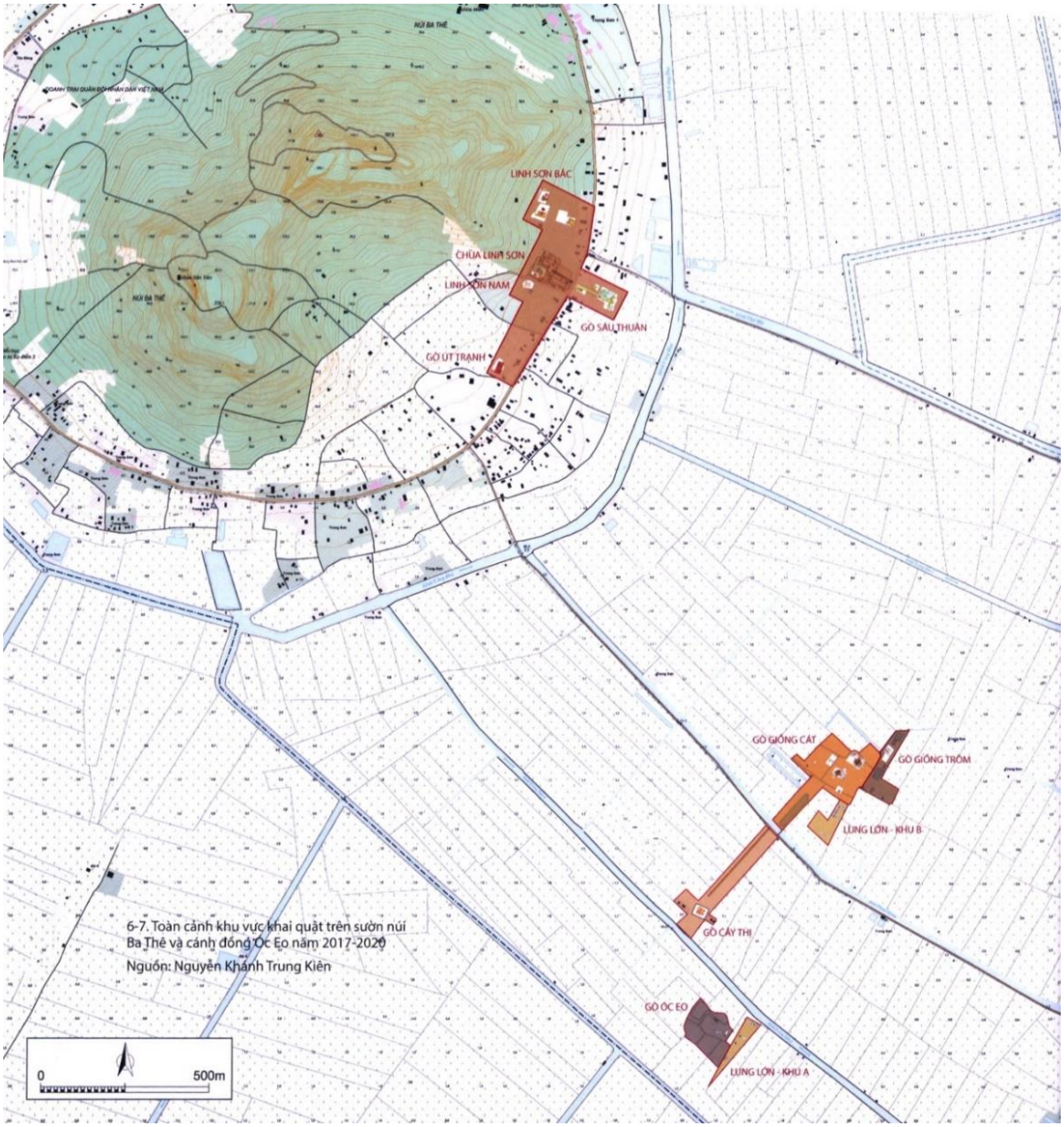

Fig. S2. Site plan of Oc Eo after the Vietnamese investigations of 2017–2020. The locations of Mount Ba The, Lung Lon ancient canal and Go Oc Eo are all marked (*14*).

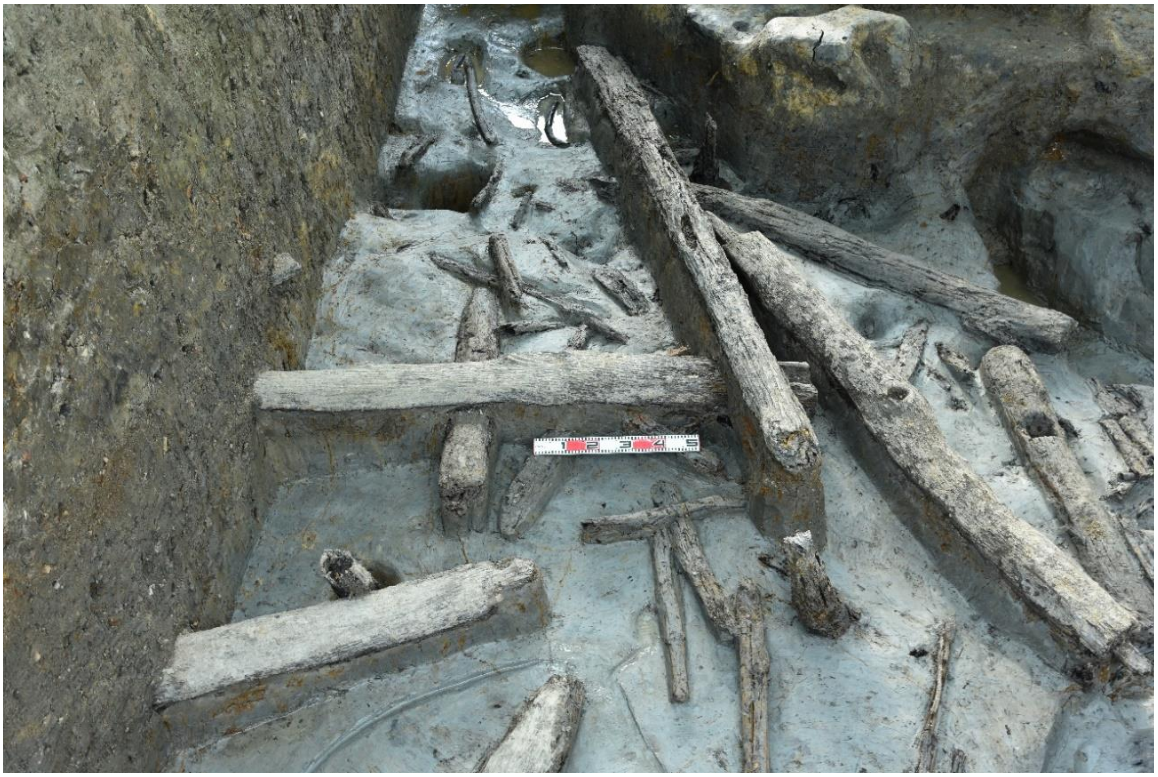

Fig. S3. Wooden posts from collapsed stilt houses in the Lung Lon Area B sector of Oc Eo.
It was thought by the excavators that these stilt houses were originally raised along an ancient canal bank (Photo source: Khanh Trung Kien Nguyen).

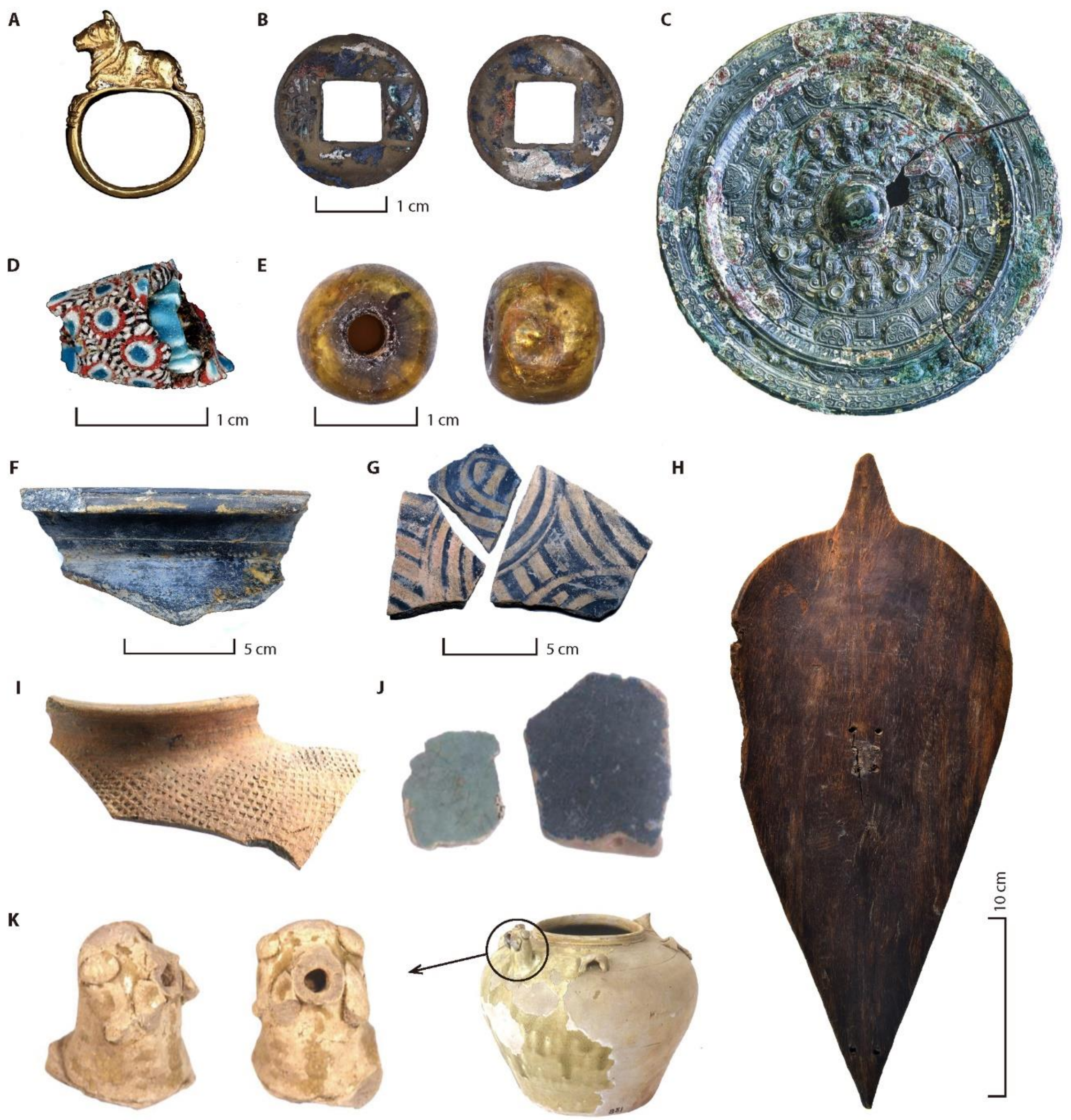

Fig. S4. Exotic artifacts surface collected or excavated in the Oc Eo site complex. (A) Gold ring with relief zebu (Nandi) decoration from Go Giong Cat B; (B) Han Dynasty Wu Zhu coin from Lung Lon; (C) Bronze mirror of Eastern Han Dynasty date from Go Giong Cat B (2nd-3rd centuries CE); (D) Roman style glass mosaic bead; (E) Roman style gold-coated glass bead, probably made in Egypt; (F) Black-slipped South Asian rim sherd from Lung Lon; (G) Painted Indian sherds; (H) Leaf-shaped wooden paddle of eastern Indonesian style from Lung Lon (1st-3rd centuries CE); (I) Paddle-impressed Han Dynasty rim sherd from Nen Chua; (J) Two blue glazed ceramic sherds from West Asia; (K) A lug from a Chinese light green glazed "chicken head" wine jar found at Nen Chua, with a complete example (at right, but not from Oc Eo) stored in the Bac Ninh Museum (*14*).

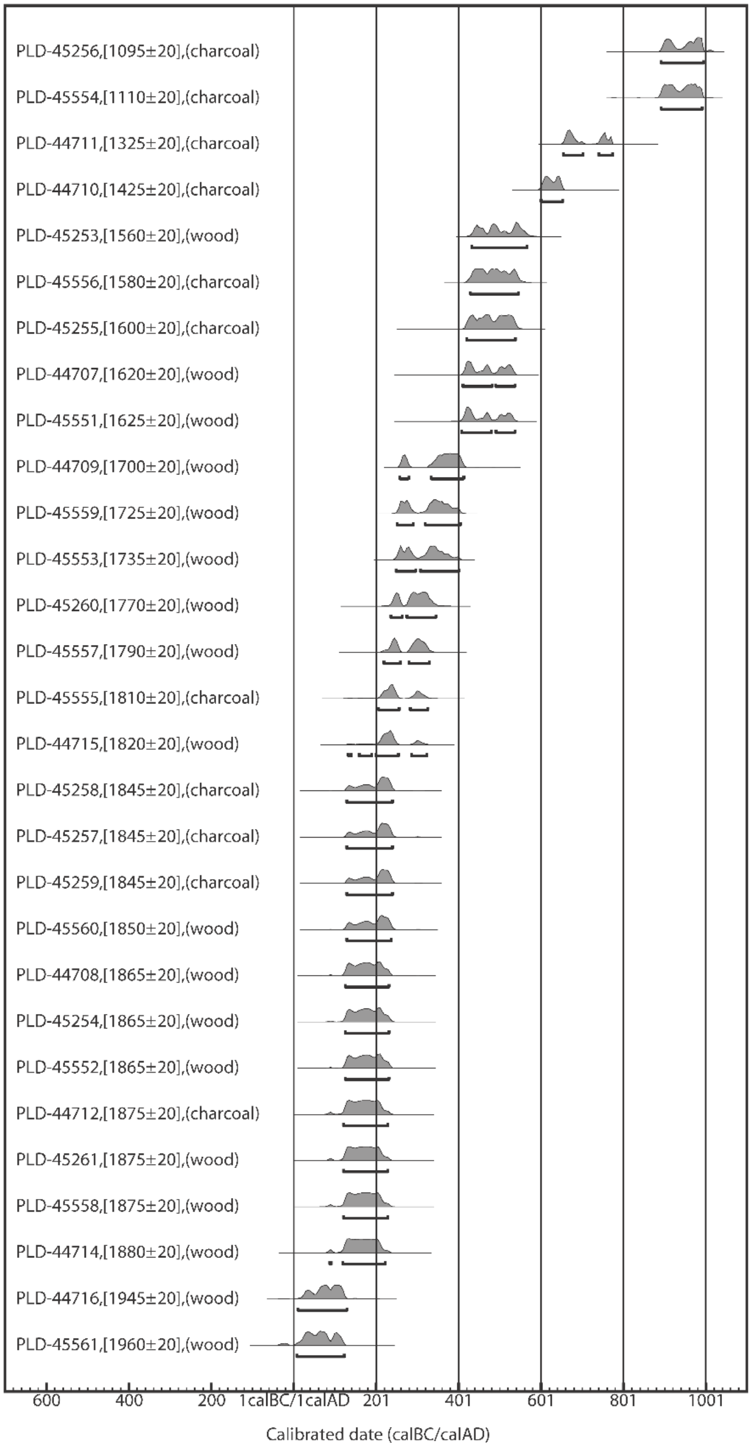

Fig. S5. Radiocarbon dates from Oc Eo.

Twenty-nine radiocarbon dates from the 2017-2020 excavations at Oc Eo (*24*), listed in Table S1, calibrated with OxCal v4.4.2 (*88*) and presented at 2σ probability.

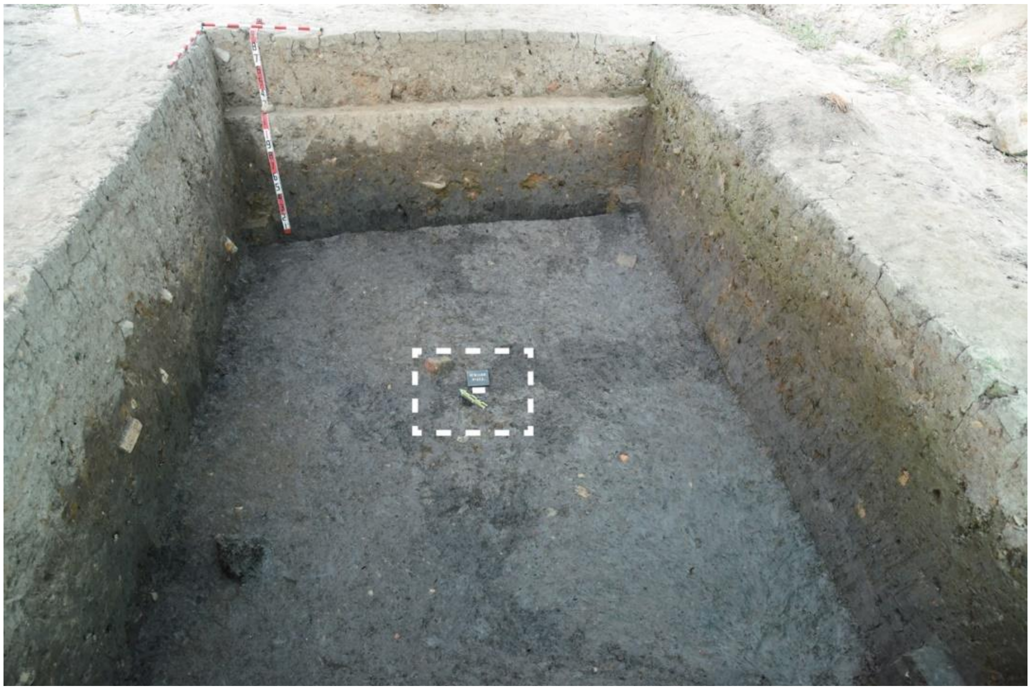

Fig. S6. The find place of the large footed grinding slab.

One of the largest footed grinding slabs (inside the dotted white rectangle, sample code: OE18.LLO.B.H1.M1.L3.3) found at Oc Eo was excavated in 2018, just above a 14C sample dated to 207–326 cal. CE. (14C laboratory code: PLD-45555) (Photo source: Khanh Trung Kien Nguyen).

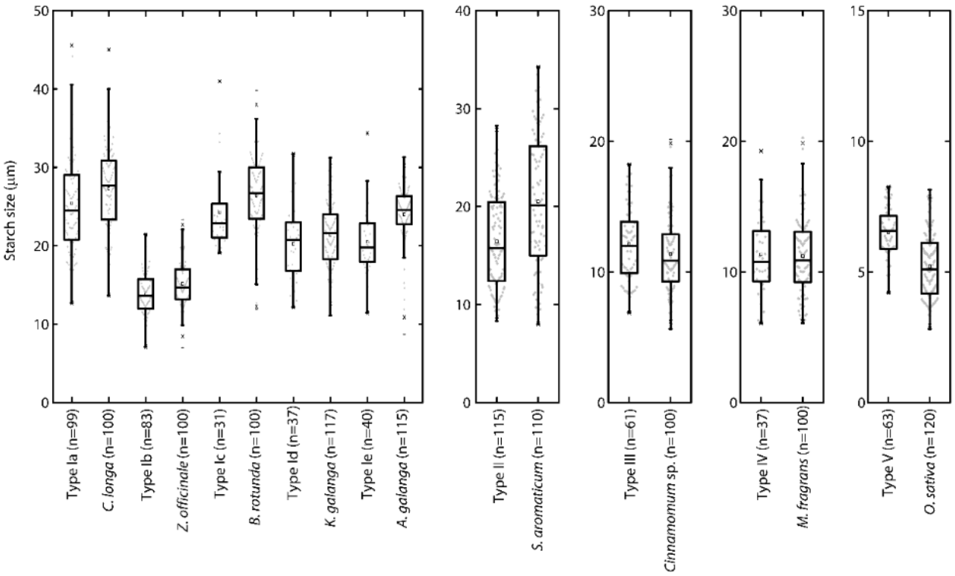

Fig. S7. Comparison of the size distributions of ancient starch grains.

Size distributions for the five major types of ancient starch grain (including the five sub-types of Type I) found at Oc Eo, compared with modern reference samples labelled by species.

Table S1. Radiocarbon dates from Oc Eo, indicative of major occupation between the 1st and 8th centuries CE. The laboratory dates are from (*24*) and have been calibrated with OxCal v4.4.2 (*88*) to 2σ cal. BC/AD.

Sample No.	Lab No.	Material	14C age (yr BP±1σ)	Cal. BC/AD (2σ)
OE18.GGC.A.H1.MG02	PLD-44708	Wedge-shaped stick - from a wooden building at HKT1 (Test pit 1)	1865±20	125-231 cal AD (95.45%)
OE18.GGC.A.H1.MG04	PLD-45254	Wedge stick - from a wooden building at HKT2 (Test pit 2)	1865±20	126-231 cal AD (95.45%)
OE18.GGC.A.H1.MG05	PLD-45552	Wooden pillar from a wooden building at HKT7 (Test pit 7)	1865±20	126-231 cal AD (95.45%)
OE19.GGC.A.H4VT.MG05	PLD-45553	Vertical wooden bar, west wall - square well structure	1735±20	250-295 cal AD (34.69%) 309-403 cal AD (60.76%)
OE19.GGC.A.H4VD.MG01	PLD-44709	Horizontal wooden bar, east wall - square well structure	1700±20	259-279 cal AD (16.22%) 336-413 cal AD (79.23%)
OE19.GGC.A.H3.MG02	PLD-44707	Wooden sample from southeast corner of the lower square wooden building	1620±20	412-481 cal AD (57.24%) 491-537 cal AD (38.21%)
OE19.GGC.A.H3.MG03	PLD-45551	Wooden sample from northeast corner of the lower square wooden building	1625±20	408-444 cal AD (37.68%) 447-481 cal AD (21.57%) 492-537 cal AD (36.20%)
OE19.GGC.A.H5L7.M1	PLD-45255	Charcoal sample from above the embankment clay layer	1600±20	422-538 cal AD (95.45%)
OE19.GGC.A.H3.MG04	PLD-45253	Wooden sample from northern wall of the lower square wooden building	1560±20	433-468 cal AD (28.00%) 473-522 cal AD (36.61%) 524-564 cal AD (30.83%)
OE19.LLO.B.H3.F19.M08	PLD-44716	Wooden sample from an architectural structure located at the bottom of Lung Lon canal	1945±20	11-129 cal AD (94.08%) 144-154 cal AD (0.92%) 194-199 cal AD (0.44%)
OE19.LLO.B.H3.ST.M10	PLD-45561	Wooden sample from an architectural structure located at the bottom of Lung Lon canal	1960±20	23- 18 cal BC (0.99%) 8-122 cal AD (94.46%)
OE19.LLO.B.H3m3.L2.M14	PLD-44714	Wooden paddle	1880±20	88- 92 cal AD (1.04%) 120-226 cal AD (94.41%)
OE19.LLO.B.H5e6.L3.11	PLD-45259	Charcoal sample on the surface of the substratum (early phase)	1845±20	129-239 cal AD (95.45%)
OE19.LLO.A.H7.ST.M02	PLD-45557	Wooden pillar associated with the Lung Lon canal	1790±20	220-259 cal AD (34.08%) 279-332 cal AD (61.37%)
OE19.LLO.A.H7.ST.M05	PLD-45558	Wooden pillar associated with the Lung Lon canal	1875±20	121-229 cal AD (95.45%)
OE19.LLO.A.H7.ST.M07	PLD-45261	Wooden rod with manufacturing traces	1875±20	123-228 cal AD (95.45%)
OE19.LLO.B.H3.F18.M05	PLD-45560	Wooden post associated with the Lung Lon canal	1850±20	129-238 cal AD (95.45%)
OE19.LLO.A.H7.ST.M01	PLD-44715	Wooden pillar associated with the Lung Lon canal	1820±20	133-137 cal AD (0.40%) 165-188 cal AD (3.26%) 201-255 cal AD (73.68%) 285-325 cal AD (18.12%)
OE18.LLO.B.H1e4.L3.10	PLD-45555	Charcoal sample from the first stage of Lung Lon canal activity	1810±20	207-256 cal AD (64.52%) 283-326 cal AD (30.93%)
OE19.LLO.B.H5c2.L3.8.M16	PLD-45260	Wooden cane	1770±20	238-262 cal AD (22.12%) 276-345 cal AD (73.33%)
OE19.LLO.B.H3.ST.M01	PLD-45559	Wooden rod with manufacturing traces	1725±20	252-291 cal AD (30.53%) 318-405 cal AD (64.92%)
OE18.LLO.H4e3.L3.3	PLD-45556	Charcoal sample from an early settlement layer along Lung Lon canal	1580±20	428-548 cal AD (95.45%)
BT18.GST.H5.L6.F24.C1	PLD-44712	Charcoal from a substratal clay layer	1875±20	123-229 cal AD (95.45%)
BT18.GST.H6.C2.L5.4	PLD-45258	Charcoal sample from yellowish clay	1845±20	129-240 cal AD (95.45%)
BT18.GST.H5.L3.5.B1.F22	PLD-45257	Charcoal sample from embankment clay	1845±20	129-241 cal AD (95.45%)
BT19.GST.H10L10.b1	PLD-44710	Charcoal sample from above a square well (black soil layer)	1425±20	602-653 cal AD (95.45%)
BT17.GST.H1L8	PLD-44711	Charcoal sample associated with a furnace (?)	1325±20	655-689 cal AD (56.34%) 697-702 cal AD (2.11%) 741-773 cal AD (37.00%)
BT17.GST.H3.L5	PLD-45554	Charcoal sample associated with an embankment	1110±20	892-992 cal AD (95.45%)
BT17.GST.H1L4	PLD-45256	Charcoal sample from below a layer of roof tiles in H1 (Excavation trench 1)	1095±20	893- 932 cal AD (37.73%) 940- 994 cal AD (57.26%) 1008-1010 cal AD (0.46%)

Table S2. Details of the 12 stone tools discussed in this study. Eight samples are stored in the An Giang Provincial Museum and four are still kept in the Oc Eo Archaeological Field Station. OD = outside diameter; ID = interior diameter.

Number	Artifacts code	Source	Tool type	Preservation condition	Length (cm)	Width (cm)	Material
1	BTAG-2191	An Giang museum	Footed grinding slab	Intact	51	23	Sandstone
2	BTAG-2193	An Giang museum	Footed grinding slab	Intact	52	22	Basalt
3	BTAG-2008-D	An Giang museum	Footed grinding slab	Intact	64	29	Sandstone
4	BTAG-4127-D	An Giang museum	Footed grinding slab	Intact	41	18	Sandstone
5	OE.19.GGC.A.H2.L5	Go Giong Cat. A	Footed grinding slab	Broken	26	24	Slate?
6	OE.19.GGC.A.H1.L8	Go Giong Cat. A	Footed grinding slab	Broken	22	23	Sandstone
7	OE.18.LLO.B.H1.M1.L3.3	Lung Lon. B	Footed grinding slab	Intact	76	31	Sandstone
8	BTAG-2187-D	An Giang museum	Muller	Intact	15	6	Ironstone?
9	BTAG-2188-D	An Giang museum	Muller	Broken	15	8	Sandstone
10	BTAG-2019-D	An Giang museum	Mortar	Broken	18.5 (OD)	10.5 (ID)	Basalt
11	OE.19.LLO.B.H3.b4.L2.7	Lung Lon. B	Mortar	Broken	11.5	11	Basalt
12	BTAG-3147-D	An Giang museum	Pestle	Broken	15	6	Sandstone

Table S3. Morphological features of the starch grains recovered from Oc Eo.

The table details shapes, size ranges, and the characteristics of hila, fissures, lamellae, and extinction crosses. Identifications are based on comparisons with modern reference samples.

Type	Granule shape	Size range (µm)	Mean size (µm)	Hilum	Protrusion	Fissures	Lamellae	Extinction cross	Identification
Ia	Triangular ovate	12.69-45.54	25.4± 6.37	Extremely eccentric	Present	Absent	Absent	Bent	*Curcuma longa*
Ib	Triangular ovate	7.11-21.44	13.77± 2.94	Extremely eccentric	Present	Absent	Absent	Bent	*Zingiber officinale*
Ic	Triangular ovate	19.1-40.98	24.27± 4.93	Extremely eccentric	Present	Absent	Visible on some grains	Bent	*Boesenbergia rotunda*
Id	Subround	12.18-31.69	20.23± 4.78	Extremely eccentric	Present	Absent	Absent	Bent	*Kaempferia galanga*
Ie	Elongate ovate	11.46-34.36	20.45± 4.15	Highly eccentric	Absent	Absent	Absent	Bent	*Alpinia galanga*
II	Ovate	8.31-28.26	16.43± 4.8	Eccentric	Absent	Absent	Visible on some grains	Bent	*Syzygium aromaticum*
III	Ovate	6.88-18.22	12.11± 2.82	Eccentric	Absent	Absent	Visible on some grains	Bent	*Cinnamomum* sp.
IV	Round/Half-round	6.08-19.25	11.31 ± 3.09	Centric	Absent	Stellate, linear fissures	Absent	Slightly bent	*Myristica fragrans*
V	Polygonal	4.2-8.25	6.52±1	Centric	Absent	Absent	Absent	Straight	*Oryza sativa*

Table S4. Major types and numbers of the phytoliths and pollen grains recovered from the Oc Eo food preparation surfaces. In total, 100 phytoliths classified into 24 types generally were produced from Poaceae (grasses, including rice), Arecaceae (palm), Musaceae (banana), Cyperaceae (sedge) and other plants; 306 Myrtaceae pollen grains were recovered from two newly excavated footed grinding slabs in Go Giong Cat.

Artifact code / Types of phytolith & pollen grain	ST-84-OE BT-AG-2191	BT-AG-2193	BTAG-2008-D	BTAG-4127-D	OE.19.GGC.A.H2.L5	OE.19.GGC.A.H1.L8	OE.18.LLO.B.H1.M1.L3.3	BTAG-2187-D	BTAG-2188-D	BTAG-2019-D	OE.19.LLO.B.H3.b4.L2.7	BTAG-3147-D
Oryza bulliform	3	17	1	2					3	3		
Double-peaked		24		1			1		3			
Scooped bilobate	1	6	1	1				1	1			
Volcaniform	11											
Spheroid echinate	4	30		5				2	45	39		5
Elongate smooth	13	10	2	14						8		10
Elongate echinate	9	23	2	2								35
Long saddle	5	6	1	12						8		12
Short saddle	3	3		3								3
Bilobate	7	10	1	3						15		9
Sponge spicules	5	9	3	6						17		8
Tracheary annulate	3	4		1						3		1
Bulliform	1	14		6			1			7		5
Square	3	12		6						5		3
Rectangle	2	8		5			1					6
acicular	2	5		1						1		
Rondel	2	1										6
Tower		5		3						1		4
Cross	1											1
Elongate crenate	2									1		
Cyperaceae achene	1	1										1
Cuneiform		1										
Elongate tabular		1										
Canarium		1										
Myrtaceae pollen					221	85						

Table S5. Major archaeological sites with identified remains of spices.

The table presents the reported spices related to this study. They were found at 27 archaeological sites located in the Mediterranean, North Africa, South Asia, East Asia, and Southeast Asia.

Site	Country	Region	Date	Sample material	Method	Spices	Reference
Farmana	India	South Asia	2600-2200 BCE	Pottery, human teeth	Starch	Turmeric, ginger	(*33*, *89*)
Megiddo	Levant	Mediterranean	ca. 1630–1550 BCE	Dental calculus	Proteomics	Turmeric	(*3*)
Motya	Italy	Mediterranean	800-500 BCE	Dental calculus	GC-MS Analysis	Turmeric	(*90*)
Quseir al-Qadim	Egypt	North Africa	50-1300 CE	Sediment	Sieving	Ginger, turmeric, cardamom, black myrobalan, fagara, betelnut, black pepper	(*8*)
Wangshanqiao	China	East Asia	ca. 300 BCE	Tomb I	Water sieving	Ginger, sichuan pepper	(*40*)
Baoshan	China	East Asia	ca. 300 BCE	Tomb II	Water sieving	Ginger, sichuan pepper	(*40*)
Wangshan	China	East Asia	ca. 300 BCE	Tomb I and II	Water sieving	Ginger, Sichuan pepper	(*40*)
Fenghuangshan	China	East Asia	ca. 160 BCE	Tomb 168	Water sieving	Ginger, sichuan pepper	(*40*)
Luobowan	China	East Asia	ca. 160 BCE	Tomb I	Water sieving	Ginger, sichuan pepper	(*40*)
Xiaojiacaochang	China	East Asia	ca. 160 BCE	Tomb 26	Water sieving	Ginger, Sichuan pepper, star anise	(*40*)
Mawangdui	China	East Asia	ca. 160 BCE	Tomb I and III	Water sieving	Lesser galangal, ginger, *C. japonicum*, sichuan pepper	(*46*)
Kantharodai	Sri Lanka	South Asia	ca. 200 BCE	Sediment	Flotation	*Alpinia* cf. *zerumbet*	(*45*)
Kirinda	Sri Lanka	South Asia	ca. 260-900 CE	Sediment	Flotation	*Alpinia* cf. *zerumbet*	(*45*)
Angkor Wat	Thailand	Southeast Asia	1000-1800 CE	Sediment	Flotation	Crepe ginger, black pepper, long pepper	(*23*)
Ta Prohm	Thailand	Southeast Asia	800-1000 CE	Sediment	Flotation	*Alpinia* sp.	(*23*)
Terqa	Syria	Mediterranean	ca.1700 BCE	Plant remains in jar	Hand-picked	Clove	(*53*, *91*)
Segaran IIA	Indonesia	Southeast Asia	160 BCE-230 CE	Sediment	Hand-picked?	Clove	(*55*)
Mantai	Sri Lanka	South Asia	500-1100 CE	Sediment	Flotation	Clove, black pepper	(*21*)
Pulau Ay site 1	Indonesia	Southeast Asia	ca. 1500-300 BCE	Potsherd	Starch	Nutmeg	(*56*)
Deir el-Bahari	Egypt	North Africa	ca. 1500 BCE	Sediment	Hand-picked?	Nutmeg	(*58*, *92*)
Yavneh	Israel	Mediterranean	ca. 900-800 BCE	Chalices	Residue analysis	Nutmeg	(*57*)
Khairadih	India	South Asia	700-200 BCE	Sediment	Flotation?	Nutmeg	(*22*, *93*)
Sanghol	India	South Asia	100-300 CE	Fire altar	Hand-picked	Nutmeg	(*94*, *95*)
Heraion of Samos	Greek	Mediterranean	700-600 BCE	Sacred precinct	Hand-picked	Chinese cinnamon?	(*64*)
Tell Qasile	Israel	Mediterranean	1100-900 BCE	Flasks	Residue analysis	Cinnamon	(58, 96)
Tel Dor	Israel	Mediterranean	1100-900 BCE	Flasks	Residue analysis	Cinnamon	(58, 96)
Kinneret	Israel	Mediterranean	1100-900 BCE	Flasks	Residue analysis	Cinnamon	(58, 96)

Table S6. Sources for the modern reference samples used in this study. Those labelled as "Australia" come from retail outlets in Canberra that specialize in Asian cuisine.

Number	Common name	Binomial name	Locality	Country	Main part used
1	Turmeric	*Curcuma longa*	An Giang	Vietnam	Rhizome
2	Fingerroot	*Boesenbergia rotunda*	An Giang	Vietnam	Rhizome
3	Galangal	*Alpinia officinarum*	An Giang	Vietnam	Rhizome
4	Ginger	*Zingiber officinale*	Ho Chi Minh	Vietnam	Rhizome
5	Sand ginger	*Kaempferia galanga*	Guangxi	China	Rhizome
6	Black pepper	*Piper nigrum*	Canberra	Australia	Seed
7	Long pepper	*Piper longum*	Canberra	Australia	Seed
8	Clove	*Syzygium aromaticum*	Canberra	Australia	Dried bud, seed
9	Nutmeg	*Myristica fragrans*	Canberra	Australia	Seed
10	Mace	*Myristica fragrans*	Canberra	Australia	Outer coating of seed
11	Coriander	*Coriandrum sativum*	Canberra	Australia	Seed
12	Lemon grass	*Cymbopogon citratus*	Canberra	Australia	Bulb
13	Cinnamon	*Cinnamomum verum*	Canberra	Australia	Inner bark
14	Chinese cassia	*Cinnamomum cassia*	Canberra	Australia	Bark
15	Cumin	*Cuminum cyminum*	Canberra	Australia	Seed
16	Fennel	*Foeniculum vulgare*	Canberra	Australia	Seed
17	Black mustard	*Brassica nigra*	Canberra	Australia	Seed
18	Yellow mustard	*Brassica hirta*	Canberra	Australia	Seed
19	Poppy	*Papaver somniferum*	Canberra	Australia	Seed
20	Curry leaf	*Murraya koenigii*	Canberra	Australia	Leaf
21	Basil	*Ocimum basilicum*	Canberra	Australia	Seed
22	Kali Jeeri	*Centratherum anthelminticum*	Canberra	Australia	Seed
23	Fenugreek	*Trigonella foenum-graecum*	Canberra	Australia	Seed
24	Green cardamom	*Elettaria cardamomum*	Canberra	Australia	Seed
25	Black cardamom	*Amomum subulatum*	Canberra	Australia	Seed
26	Star anise	*Illicium verum*	Canberra	Australia	Seed
27	Harar	*Terminalia chebula*	Canberra	Australia	Seed
28	Cuddapah almond	*Buchanania lanzan*	Canberra	Australia	Seed
29	Eggplant	*Solanum melongena*	Canberra	Australia	Fruit
30	Mango	*Mangifera indica*	Canberra	Australia	Fruit
31	Coconut	*Cocos nucifera*	Canberra	Australia	Fruit
32	Banana	*Musa* sp.	Ho Chi Minh	Vietnam	Fruit

REFERENCES AND NOTES

1. A. Dalby, *Dangerous Tastes: The Story of Spices* (University of California Press, 2000).

2. P. N. Ravindran, *The Encyclopedia of Herbs and Spices* (CABI, 2017).

3. A. Scott, R. C. Power, V. Altmann-Wendling, M. Artzy, M. A. Martin, S. Eisenmann, R. Hagan, D. C. Salazar-García, Y. Salmon, D. Yegorov, Exotic foods reveal contact between South Asia and the Near East during the second millennium BCE. *Proc. Natl. Acad. Sci. U.S.A.* **118**, e2014956117 (2021).

4. A. Plu, Bois et graines, in *La Momie de Ramsès II: Contribution Scientifique à l'Égyptologie,* L. Balout, C. Roubet, Eds. (Recherches sur les Civilisations, 1985), pp. 166–174.

5. I. G. Giacosa, *A Taste of Ancient Rome* (The University of Chicago Press, 1994).

6. G. Prance, M. Nesbitt, *The Cultural History of Plants* (Routledge, 2012).

7. G. K. Young, *Rome's Eastern Trade: International Commerce and Imperial Policy 31 BC-AD 305* (Routledge, 2001).

8. M. Van der Veen, J. Morales, The Roman and Islamic spice trade: New archaeological evidence. *J. Ethnopharmacol.* **167**, 54–63 (2015).

9. L. W. Leng, C. S. Choo, Maritime Southeast Asia in global trade in pre-modern times: A historical geography perspective. *J. Marit. Coast. Stud.* **1**, 1–15 (2019).

10. M. T. Stark, Inscribing legitimacy and building power in the Mekong delta, in *Counternarratives and Macrohistories: New Agendas in Archaeology and Ancient History*, G. Emberling, Ed. (Cambridge Univ. Press, 2015), pp. 75–105.

11. A. O. Zakharov, State formation in first millennium Southeast Asia: A reappraisal. *Soc. Evol. Hist.* **18**, 217–240 (2019).

12. M. Vickery, Funan reviewed: Deconstructing the ancients. *Bull.Ec. Fr. Extr. Orient.* **90**, 101–

143 (2003).

13. A. K. Carter, L. Dussubieux, M. T. Stark, H. A. Gilg, Angkor Borei and protohistoric trade networks: A view from the glass and stone bead assemblage. *Asian Perspect.* **60**, 32–70 (2020).

14. Vietnam Academy of Social Sciences, in *New Archaeological Findings of Oc Eo Culture at Oc Eo-Ba The Complex 2017-2020*, M. T. Bui, G. D. Nguyen, K. T. K. Nguyen, Eds. (Social Science Publishing House, 2022).

15. P. Indrawooth, Dvaravati: Early buddhist kingdom in central Thailand, in *Indo-Thai Historical and Cultural Linkages*, N. Misra, S. Sahain, Eds. (Manohar, 2007), pp. 37–64.

16. H.-J. Weisshaar, Legged saddle querns of South Asia, in *Zeitschrift für Archäologie Aussereuropäischer Kulturen*, B. Vogt, J. Eiwanger, Eds. (Reichert Verlag, 2014), vol. **6**, pp. 119–144.

17. R. Muthucumarana, A. S. Gaur, W. M. Chandraratne, M. Manders, B. Ramlingeswara Rao, R. Bhushan, V. D. Khedekar, A. M. A. Dayananda, An early historic assemblage offshore of Godawaya, Sri Lanka: Evidence for early regional seafaring in South Asia. *J. Marit. Archaeol.* **9**, 41–58 (2014).

18. C. T. Sen, *Curry: A Global History* (Reaktion Books, 2009).

19. P. J. Cherian, Pattanam archaeological site: Evidence of maritime exchanges. *Tamil Civilis.* **12**, 22–31 (2012).

20. M. Rahman, C. C. Castillo, C. Murphy, S. M. Rahman, D. Q. Fuller, Agricultural systems in Bangladesh: The first archaeobotanical results from early historic Wari-Bateshwar and early medieval Vikrampura. *Archaeol. Anthropol. Sci.* **12**, 37 (2020).

21. E. Kingwell-Banham, W. Bohingamuwa, N. Perera, G. Adikari, A. Crowther, D. Q. Fuller, N. Boivin, Spice and rice: Pepper, cloves and everyday cereal foods at the ancient port of Mantai, Sri Lanka. *Antiquity* **92**, 1552–1570 (2018).

22. T. J. Zumbroich, From mouth fresheners to erotic perfumes: The evolving socio-cultural significance of nutmeg, mace and cloves in South Asia. *ejournal Indian Med.* **5**, 37–97 (2012).

23. C. C. Castillo, A. Carter, E. Kingwell-Banham, Y. Zhuang, A. Weisskopf, R. Chhay, P. Heng, D. Q. Fuller, M. Stark, The Khmer did not live by rice alone: Archaeobotanical investigations at Angkor Wat and Ta Prohm. *Archaeol. Res. Asia* **24**, 100213 (2020).

24. K. T. K. Nguyen, M. Yamagata, S. Kubo, Based on new analysis result to date Oc Eo-Ba The archaeological sites. *Archaeology* **6,** 12–27 (2022).

25. P. Changmai, R. Pinhasi, M. Pietrusewsky, M. T. Stark, R. M. Ikehara-Quebral, D. Reich, P. Flegontov, Ancient DNA from Protohistoric Period Cambodia indicates that South Asians admixed with local populations as early as 1st-3rd centuries CE. *Sci. Rep.* **12**, 22507 (2022).

26. D. R. Piperno, E. Weiss, I. Holst, D. Nadel, Processing of wild cereal grains in the Upper Palaeolithic revealed by starch grain analysis. *Nature* **430**, 670–673 (2004).

27. X. Yang, Z. Wan, L. Perry, H. Lu, Q. Wang, C. Zhao, J. Li, F. Xie, J. Yu, T. Cui, Early millet use in northern China. *Proc. Natl. Acad. Sci. U.S.A.* **109**, 3726–3730 (2012).

28. D. R. Piperno, A. J. Ranere, I. Holst, P. Hansell, Starch grains reveal early root crop horticulture in the Panamanian tropical forest. *Nature* **407**, 894–897 (2000).

29. D. R. Piperno, A. J. Ranere, I. Holst, J. Iriarte, R. Dickau, Starch grain and phytolith evidence for early ninth millennium B.P. maize from the Central Balsas River Valley, Mexico. *Proc. Natl. Acad. Sci. U.S.A.* **106**, 5019–5024 (2009).

30. R. Fullagar, J. Field, T. Denham, C. Lentfer, Early and mid Holocene tool-use and processing of taro (*Colocasia esculenta*), yam (*Dioscorea* sp.) and other plants at Kuk Swamp in the highlands of Papua New Guinea. *J. Archaeol. Sci.* **33**, 595–614 (2006).

31. W. Wang, K. D. Nguyen, H. Dang Le, C. Zhao, M. T. Carson, X. Yang, H.-c. Hung, Before rice and the first rice: Archaeobotanical study in Ha Long Bay, northern Vietnam. *Front.*

Earth Sci. **10**, 881104 (2022).

32. K. P. Nair, *Turmeric (Curcuma longa L.) and Ginger (Zingiber officinale Rosc.)-World's Invaluable Medicinal Spices: The Agronomy and Economy of Turmeric and Ginger* (Springer, 2019).

33. A. Kashyap, S. A. Weber, Harappan plant use revealed by starch grains from Farmana, India. *Antiq. Proj. Gallery* **84**, 326 (2010).

34. R. C. Thompson, *A Dictionary of Assyrian Botany* (British Academy, 1949).

35. D. E. Sopher, Indigenous uses of turmeric (*Curcuma domestica*) in Asia and Oceania. *Anthropos* **59**, 93–127 (1964).

36. P.-Y. Manguin, The transmission of Vaiṣṇavism across the Bay of Bengal: Trade networks and state formation in early historic Southeast Asia, in *Early Global Interconnectivity Across the Indian Ocean World, Volume II: Exchange of Ideas, Religions and Technologies*, A. Schottenhammer, Ed. (Palgrave McMillan, 2019), pp. 51–68.

37. P. N. Ravindran, K. N. Babu, *Ginger: The Genus Zingiber* (CRC Press, 2016).

38. J. O'Connell, *The Book of Spice: From Anise to Zedoary* (Profile Books, 2015).

39. D. L. Wu, Study on origin of ginger. *Agric. Archaeol.* **2**, 247–250 (1985).

40. P. Sheng, H. Zhou, J. Liu, H. Jiang, Some like it hot: Sichuan pepper (*Zanthoxylum bungeanum*) and other spices from a late Bronze Age kingdom (Chu State) in Hubei, China, *Archaeol. Anthropol. Sci.* **12**, 249 (2020).

41. P. N. Ravindran, G. S. Pillai, I. Balachandran, M. Divakaran, Galangal, in *Handbook of Herbs and Spices: Volume 2*, K. V. Peter, Ed. (Woodhead Publishing, ed. 2, 2012), pp. 303–318.

42. P. N. Ravindran, G. S. Pillai, Galanga, in *Handbook of Herbs and Spices: Volume 3*, K. V. Peter, Ed. (Woodhead Publishing, 2006), pp. 347–356.

43. J. Seidemann, *World Spice Plants: Economic Usage, Botany, Taxonomy* (Springer, 2005).

44. A. Kumar, Phytochemistry, pharmacological activities and uses of traditional medicinal plant *Kaempferia galanga* L.- an overview. *J. Ethnopharmacol.* **253**, 112667 (2020).

45. C. Murphy, A. Weisskopf, W. Bohingamuwa, G. Adikari, N. Perera, J. Blinkhorn, M. Horton, D. Q. Fuller, N. Boivin, Early agriculture in Sri Lanka: New archaeobotanical analyses and radiocarbon dates from the early historic sites of Kirinda and Kantharodai (Kandarodai). *Archaeol. Res. Asia* **16**, 88–102 (2018).

46. The Hunan Provincial Museum, The Institute of Archaeology of Hunan Province, *Tombs 2 and 3 of the Han Dynasty at Mawangdui, Changsha Report on Excavation Volume I* (Cultural Relics Publishing House, 2004).

47. B. Pickersgill, Spices, in *The Cultural History of Plants*, G. Prance, M. Nesbitt, Eds. (Routledge, 2012), pp. 153–172.

48. Y. C. Li, H. Ji, X. H. Li, H. X. Zhang, H. T. Li, Isolation of nematicidal constituents from essential oil of *Kaempferia galanga* L rhizome and their activity against *Heterodera avenae* Wollenweber. *Trop. J. Pharm. Res.* **16**, 59–65 (2017).

49. M. Yonna, H. Fadliyah, E. Meiyanto, Fingerroot (*Boesenbergia pandurata*): A prospective anticancer therapy. *Indones. J. Cancer Chemoprevention* **9**, 102–109 (2018).

50. A. Reid, *Southeast Asia in the Age of Commerce, 1450–1680: Volume 2, Expansion and Crisis* (Yale Univ. Press, 1993).

51. R. S. Bown, *Merchant Kings: When Companies Ruled the World, 1600–1900* (St. Martin's Press, 2010).

52. J. I. Miller, *Spice Trade of The Roman Empire, 29 BC to AD 641* (Clarendon Press, 1969).

53. G. Buccellati, M. K. Buccellati, Terqa: The first eight seasons. *Les Ann. Archeol. Arabes Syriennes* **33**, 47–67 (1983).

54. M. Spriggs, Research questions in Maluku archaeology. *Cakalele* **9**, 51–64 (1998).

55. P.-Y. Manguin, A. Indradjaja, The Batujaya site: New evidence of early Indian influence in West Java, in *Early Interactions between South and Southeast Asia: Reflections on Cross-Cultural Exchange*, P.-Y. Manguin, A. Mani, G. Wade, Eds. (ISEAS Publishing, 2011), pp. 113–136.

56. P. Lape, E. Peterson, D. Tanudirjo, New data from an open Neolithic site in eastern Indonesia. *Asian Perspect.* **57**, 222–243 (2018).

57. D. Namdar, R. Neumann, S. Weiner, Residue Analysis of Chalices from the Repository Pit in *Yavneh I: The Excavation of the "Temple Hill" Repository Pit and the Cult Stands*, R. Kletter, I. Ziffer, W. Zwickel, Eds. (Academic Press, 2010), pp. 167–173.

58. A. Gilboa, D. Namdar, On the beginnings of South Asian spice trade with the Mediterranean region: A review. *Radiocarbon* **57**, 265–283 (2015).

59. G. M. Lin, Y. H. Chen, P. L. Yen, S. T. Chang, Antihyperglycemic and antioxidant activities of twig extract from *Cinnamomum osmophloeum*. *J. Tradit. Complement. Med.* **6**, 281–288 (2016).

60. L. Suriyagoda, A. J. Mohotti, J. K. Vidanarachchi, S. P. Kodithuwakku, M. Chathurika, P. C. G. Bandaranayake, A. M. Hetherington, C. K. Beneragama, "Ceylon cinnamon": Much more than just a spice. *Plants People Planet* **3**, 319–336 (2021).

61. P. N. Ravindran, K. Nirmal-Babu, M. Shylaja, *Cinnamon and Cassia: The Genus Cinnamomum* (CRC Press, 2003).

62. S. Raghavan, *Handbook of Spices, Seasonings, and Flavorings* (CRC Press, ed. 2, 2006).

63. B. B. Baumann, The botanical aspects of ancient Egyptian embalming and burial. *Econ. Bot.* **14**, 84–104 (1960).

64. S. G. Haw, Cinnamon, cassia and ancient trade. *J. Anc. Hist. Archaeol.* **4**, 5–18 (2017).

65. R. Dasanayake, Cinnamon: A spice of an Indigenous origin- historical study. *Narrations* **1**, 46–58 (2016).

66. S. E. Sidebotham, *Roman Economic Policy in the Erythra Thalassa: 30 B.C.-A.D. 217* (Brill, 1986).

67. C. Brand, E. A. Mitchell, *Holman Illustrated Bible Dictionary* (B & H Publishing Group, 2015), pp. 296.

68. P.-Y. Manguin, The rchaeology of Funan in the Mekong River delta: The Oc Eo Culturec of Vietnam, in *Arts of Ancient Vietnam: From River Plain to Open Sea*, N. Tingley, Ed. (Yale Univ. Press, 2009), pp. 100–118.

69. P.-Y. Manguin, M. T. Stark, Mainland Southeast Asia's earliest kingdoms and the case of "Funan", in *The Oxford Handbook of Early Southeast Asia*, C. F. W. Higham, C. N. Kim, Eds. (Oxford Univ. Press, 2022).

70. M. N. A. Raji, S. Ab Karim, F. A. C. Ishak, M. M. Arshad, Past and present practices of the Malay food heritage and culture in Malaysia. *J. Ethn. Foods* **4**, 221–231 (2017).

71. P. N. Ravindran, G. S. Pillai, M. Divakaran, Other Herbs and Spices: Mango Ginger to Wasabi, in *Handbook of Herbs and Spices*, K. V. Peter, Ed. (Woodhead Publishing, ed. 2, 2012), pp. 557–582.

72. X. Yang, W. Wu, L. Perry, Z. Ma, O. Bar-Yosef, D. J. Cohen, H. Zheng, Q. Ge, Critical role of climate change in plant selection and millet domestication in North China. *Sci. Rep.* **8**, 7855 (2018).

73. B. P. Jackson, D. W. Snowdon, *Atlas of Microscopy of Medicinal Plants, Culinary Herbs and Spices* (Belhaven Press, 1990).

74. Chinese Pharmacopeia Commission, *An Illustrated Handbook on Microscopic Identification of Chinese Crude Drugs for Chinese Pharmacopoeia* (People's Medical Publishing House, 2009).

75. Z. Deng, H.-c. Hung, M. T. Carson, A. A. Oktaviana, B. Hakim, T. Simanjuntak, Validating earliest rice farming in the Indonesian Archipelago. *Sci. Rep.* **10**, 10984 (2020).

76. T. Ball, K. Chandler-Ezell, R. Dickau, N. Duncan, T. C. Hart, J. Iriarte, C. Lentfer, A. Logan, H. Lu, M. Madella, D. M. Pearsall, D. R. Piperno, A. M. Rosen, L. Vrydaghs, A. Weisskopf, J. Zhang, Phytoliths as a tool for investigations of agricultural origins and dispersals around the world. *J. Archaeol. Sci.* **68**, 32–45 (2016).

77. International Committee for Phytolith Taxonomy (ICPT), International code for phytolith nomenclature (ICPN) 2.0. *Ann. Bot.* **124**, 189–199 (2019).

78. S. C. Chew, *The Southeast Asia Connection: Trade and Polities in the Eurasian World Economy, 500 BC-AD 500* (Berghahn Books, 2018).

79. H.-c. Hung, C.-y. Chao, Taiwan's Early Metal Age and Southeast Asian trading systems. *Antiquity* **90**, 1537–1551 (2016).

80. L. Malleret, *L'archéologie du Delta du Mékong, Tome Premier: L'exploration archéologique et les fouilles d'Oc-Èo* (École Francaise d'Extrême-Orient, 1959).

81. L. Malleret, *L'archéologie du Delta du Mékong, Tome Second: La civilisation matérielle d'Oc-Éo* (École Francaise d'Extrême-Orient, 1960).

82. P.-Y. Manguin, S. K. Vo, Excavations at the Ba The/ Oc Eo complex (Viet Nam), a preliminary report on the 1998 campaign, in *Southeast Asian Archaeology 1998: Proceedings of the 7th International Conference of the European Association of Southeast Asian Archaeologists, Berlin, 1998*, W. Lobo, S. Reimann, Eds. (Centre for Southeast Asian Studies, University of Hull, 2000), pp. 107–122.

83. D. V. Thang, V. V. Sen, Recognition of Oc Eo culture relic in Thoai Son District an Giang Province, Vienam. *Am. Sci. Res. J. Eng. Technol. Sci.* **36**, 271–293 (2017).

84. B. Borell, Vietnam und die maritime Seidenstrasse in den frühen Jahrhunderten nach Chr, in *Schätze der Archäologie Vietnams* (Nünnerich-Asmus Verlag & Media, 2016), pp. 125–136.

85. A. H. Thornhill, G. S. Hope, L. A. Craven, M. D. Crisp, Pollen morphology of the Myrtaceae. Part 1: Tribes Eucalypteae, Lophostemoneae, Syncarpieae, Xanthostemoneae and subfamily Psiloxyloideae. *Aust. J. Bot.* **60**, 165–199 (2012).

86. A. H. Thornhill, G. S. Hope, L. A. Craven, M. D. Crisp, Pollen morphology of the Myrtaceae. Part 2: Tribes Backhousieae, Melaleuceae, Metrosidereae, Osbornieae and Syzygieae. *Aust. J. Bot.* **60**, 200–224 (2012).

87. K. Reinhard, K. B. Lynch, A. Larsen, B. Adams, L. Higley, M. M. do Amaral, J. Russ, Y. Zhou, D. Lippi, J. Morrow, Pollen evidence of medicine from an embalming jar associated with Vittoria della Rovere, Florence, Italy. *J. Archaeol. Sci. Rep.* **21**, 238–242 (2018).

88. C. Bronk Ramsey, Bayesian analysis of radiocarbon dates. *Radiocarbon* **51**, 337–360 (2009).

89. S. A. Weber, A. Kashyap, L. Mounce, Archaeobotany at Farmana: New insights into Harappan plant use strategies, in *Excavations at Farmana, District Rohtak, Haryana, India, 2006–8*, V. Shinde, T. Osada, M. Kumar, Eds. (Research Institute for Humanity and Nature, 2011), pp. 808–823.

90. A. D'Agostino, A. Canini, G. Di Marco, L. Nigro, F. Spagnoli, A. Gismondi, Investigating plant micro-remains embedded in dental calculus of the Phoenician inhabitants of Motya (Sicily, Italy). *Plan. Theory* **9**, 1395 (2020).

91. J. Turner, *Spice: The History of A Temptation* (HarperCollins, 2004).

92. T. Hoogervorst, Chapetr 8.4: Tracing Maritime Connections between Island Southeast Asia and the Indian Ocean World, in *The Routledge Handbook of Archaeology and Globalization*, T. Hodos, Ed. (Routledge, 2016), pp. 775–791.

93. K. S. Saraswat, D. C. Saini, M. K. Sharma, S. Chanchala, Palaeobotanical and pollen analytical investigations. *Ind. Archaeol. Rev.* ***1985-6***, 122–125 (1990).

94. E. Asouti, D. Q. Fuller, *Trees and Woodlands of South India: Archaeological Perspectives* (Left Coast Press, 2008).

95. K. S. Saraswat, A. K. Pokharia, On the remains of botanical material used in fire-sacrifice ritualized during Kushana period at Sanghol (Punjab). *Pragdhara* **8**, 149–181 (1998).

96. D. Namdar, A. Gilboa, R. Neumann, I. Finkelstein, S. Weiner, Cinnamaldehyde in early Iron Age phoenician flasks raises the possibility of Levantine trade with South East Asia. *Mediterr. Archaeol. Archaeom.* **13**, 1–19 (2013).
